# Postsynthetic Transformation of Imine- into Nitrone-Linked Covalent Organic Frameworks for Atmospheric Water Harvesting at Decreased Humidity

**DOI:** 10.1021/jacs.3c02572

**Published:** 2023-05-26

**Authors:** Lars Grunenberg, Gökcen Savasci, Sebastian T. Emmerling, Fabian Heck, Sebastian Bette, Afonso Cima Bergesch, Christian Ochsenfeld, Bettina V. Lotsch

**Affiliations:** † Max Planck Institute for Solid State Research, Heisenbergstraße 1, 70569 Stuttgart, Germany; ‡ Department of Chemistry, Ludwig-Maximilians-Universität (LMU), Butenandtstraße 5-13, 81377 Munich, Germany; § e-conversion, Lichtenbergstraße 4a, 85748 Garching, Germany

## Abstract

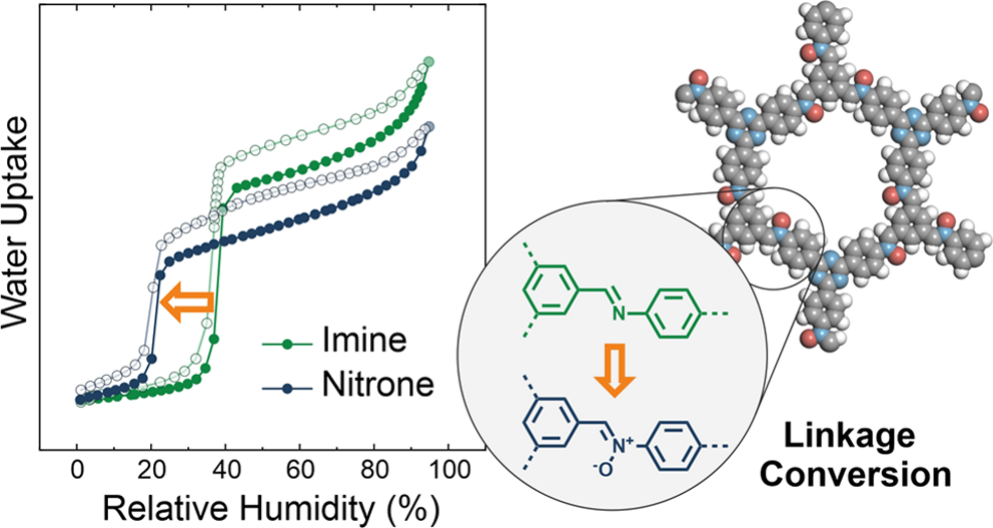

Herein, we report a facile postsynthetic linkage conversion method giving synthetic access to nitrone-linked covalent organic frameworks (COFs) from imine- and amine-linked COFs. The new two-dimensional (2D) nitrone-linked covalent organic frameworks, NO-PI-3-COF and NO-TTI-COF, are obtained with high crystallinity and large surface areas. Nitrone-modified pore channels induce condensation of water vapor at 20% lower humidity compared to their amine- or imine-linked precursor COFs. Thus, the topochemical transformation to nitrone linkages constitutes an attractive approach to postsynthetically fine-tune water adsorption properties in framework materials.

## Introduction

In recent years, covalent organic frameworks (COFs) have received increasing attention as sorbents for water vapor from the atmosphere.^1−7^ Similar to already well-established materials such as metal–organic frameworks,^8,9^ COFs offer ideal structural capabilities for this application, given their typically large specific surface areas and permanent porosity.^10^ The structural diversity of COF building blocks and linkages, coupled with their regular ordering in the crystalline material, also allow for almost infinite variation and optimization possibilities, and provide the basis for application-targeted engineering of these materials.^11−14^ Despite this adaptability of their structures, water capture in many evaluated COF systems, especially in those with pore diameters larger than 1.5 nm, is typically characterized by high uptake pressures and large hysteresis, limiting their potential for application as water harvesting materials.^15^ This implies that the water uptake performance of many existing COFs often cannot compete with the best-in-class MOFs, although their variety in building blocks, and in particular linkage composition, and the fact that COFs are not based on potentially toxic, or cost-prohibitive metals, offer great potential.^7^ Thus, several approaches have been made to improve the sorption properties of COFs. For example, the change in hydrophilicity of the pore channel surface in 2D COFs was investigated as a function of the chemical structure of COF building blocks, the linkers. Here, hydroxyl or nitro groups in the chemical structure of the linkers resulted in improved water uptake at lower relative pressures, as presented for small-pore materials with ketoenamine linkages.^6,16^ Likewise, an isoreticular series of hydroxy-functionalized azine-linked COFs showed a shift in the steep uptake region of the sorption isotherm to lower relative humidities.^5^ Uptake at low relative pressures (i.e., low humidity) is particularly desirable for water harvesting materials, and should ideally occur between 10 and 30% relative humidity.^9^ Besides functional surface groups on the pore channels, the framework topology and the pore diameter were also shown to modulate water adsorption properties. Multivalent linker combinations affording microporous trigonal^15^ or voided square-lattice^17^ materials with small pore diameters support the condensation of water and thus exhibit attractive water sorption properties.

While these concepts are generally based on the modification of the chemical structure of the building blocks, which is always related to a bottom-up or *de novo* synthesis of new COFs, postsynthetic strategies targeting the connectivity (i.e., linkage) in existing frameworks have rarely been addressed as systematic approaches to improve water adsorption properties.^7^

**Figure 1:**
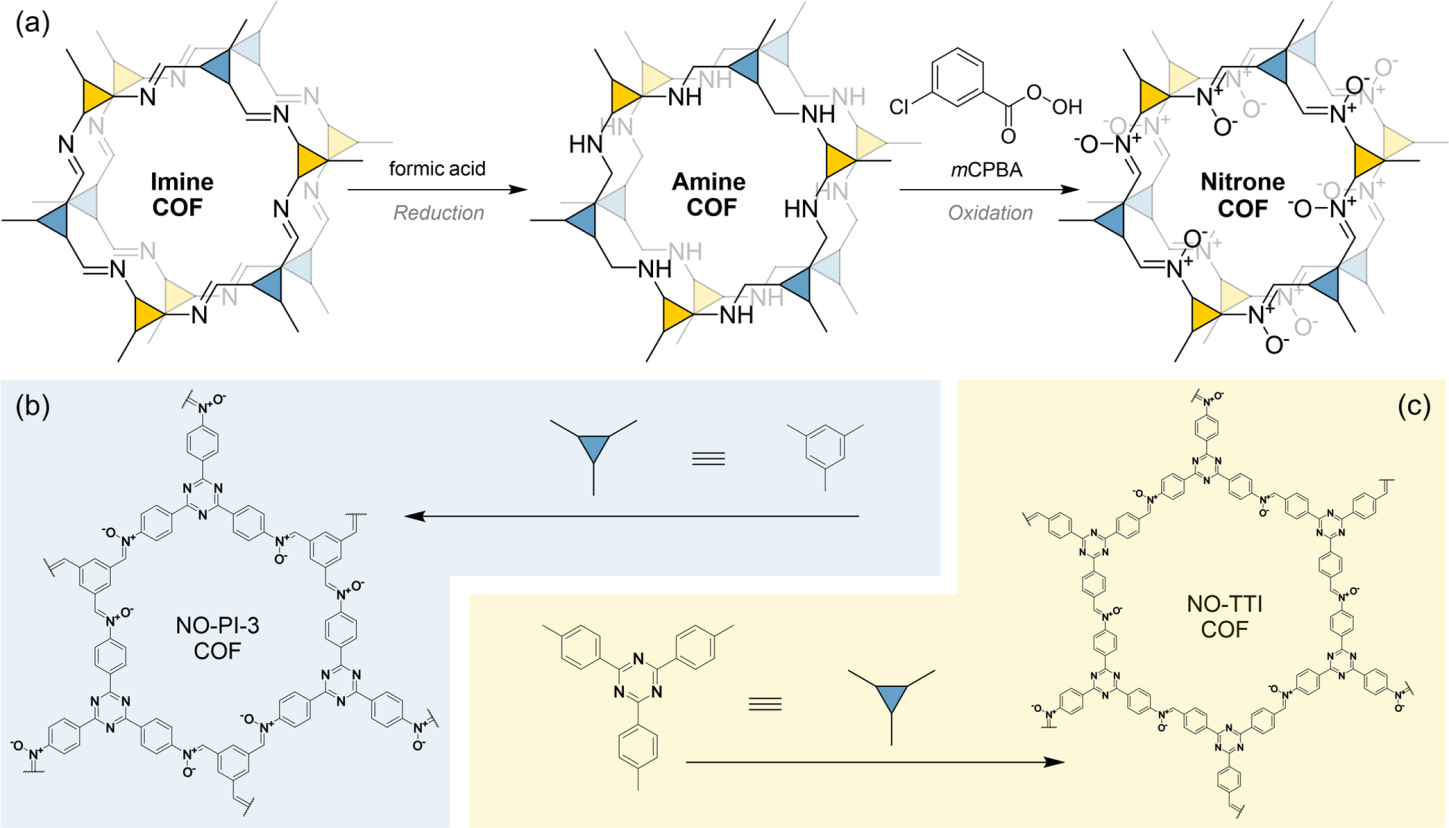
(a) Synthesis scheme for the postsynthetic transformation of imine- into nitrone-linked covalent organic frameworks. (b) Chemical structure of a single pore of (b) NO-PI-3-COF and (c) NO-TTI-COF.

Herein, we present a new postsynthetic linkage conversion protocol, applicable to imine- and amine-linked COFs, to obtain a novel class of nitrone-linked COFs with improved water adsorption capabilities at reduced humidity (Figure 1a). Compared to earlier reports, our method constitutes a selective top-down strategy to imprint desired properties into these frameworks through an atom-precise topochemical modification of their chemical structure. Our approach circumvents tedious optimization of crystallization conditions and costly material losses of precious building blocks, typically associated with *de novo* synthesis of COFs.

To demonstrate the generality of our postsynthetic oxidation method, we apply this novel approach to the synthesis of two hexagonal COFs with different pore diameters, namely, NO-PI-3-COF (1.6 nm) and NO-TTI-COF (2.1 nm), from their imine-linked parent materials (Figure 1b,c). We characterize changes in the material by utilizing a comprehensive suite of analytical techniques including Fourier transform infrared (FT-IR) spectroscopy, ^13^C and ^15^N solid-state nuclear magnetic resonance spectroscopy (ssNMR), and X-ray powder diffraction (XRPD), and correlate the COFs’ ability to adsorb water vapor, assessed by water vapor sorption experiments, to the targeted modification of the linkage chemistry.

## Results

### Material Synthesis and Analysis

As a model system for our reaction sequence, we first synthesized the imine-linked PI-3-COF from 1,3,5-triformyl benzene (TFB) and 4,4′,4″-(1,3,5-triazine-2,4,6-triyl)trianiline (TTA) under solvothermal conditions, according to our previously reported method (see details in the Supporting Information).^18^ In a following step, the imine-linked PI-3-COF was reduced to its amine-linked derivative rPI-3-COF using formic acid, following our recently developed protocol.^18^ In a second postsynthetic linkage conversion, we employed m-chloroperoxybenzoic acid (mCPBA) to oxidize secondary amine linkages in rPI-3-COF to nitrone linkages.

Upon reaction of rPI-3-COF with mCPBA, characteristic secondary amine vibrations at ν_N–H_ = 3405 cm^–1^ in the Fourier transform infrared (FT-IR) spectrum disappeared, suggesting a conversion of the linkage in the material (Figure S1). Concomitant changes of relative intensities in the fingerprint region of the spectrum, among which a very weak vibration at ν_N–O_ = 1079 cm^–1^, indicate successful oxidation to nitrone linkages.^19^ Notably, relative intensities of apparent vibrations in the spectrum of NO-PI-3-COF differ from those visible for the parent imine-linked PI-3-COF and exclude that secondary amines were simply reoxidized to imines (Figure S1).

**Figure 2:**
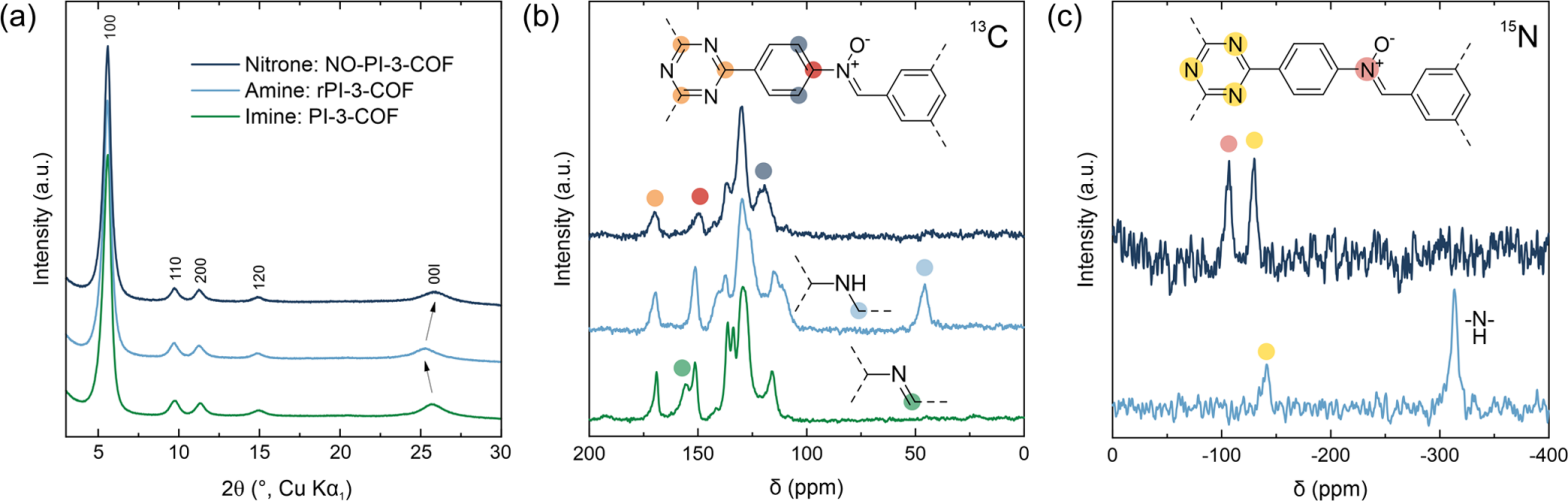
(a) XRPD pattern comparison of PI-3 (green), rPI-3 (blue), and NO-PI-3-COF (dark blue) obtained from multistep linkage conversion. As indicated by arrows, the position of the stacking reflection shifts depending on the linkage type in the framework. (b) ^13^C CP-MAS ssNMR spectra of these materials with characteristic signals highlighted. (c) ^15^N CP-MAS ssNMR spectra of rPI-3 and NO-PI-3-COF.

The ^13^C cross-polarization magic angle spinning (CP-MAS) solid-state NMR (ssNMR) spectrum of NO-PI-3-COF further corroborates this finding, lacking a characteristic imine carbon signal at δ = 155.3 ppm (PI-3-COF) and signals for the benzylic CH_2_ carbon of rPI-3-COF at δ = 45.4 ppm (Figure 2b). The ^15^N ssNMR spectrum of NO-PI-3-COF shows distinct signals at −106.3 ppm for the nitrone and at −129.4 ppm for the triazine nitrogen (Figure 2c). The absence of the imine and amine nitrogens at −57.4 ppm (Figure S17) and −313.3 ppm (Figure 2c), respectively, suggest a quantitative oxidation of amine linkages to nitrones in NO-PI-3-COF. Likewise, the data show that the oxidation treatment did not affect the nitrogen atoms of the triazine ring, and prove a selective oxidation of the linkages. The observed ssNMR chemical shifts are in line with values obtained by quantum-chemical calculations of representative molecular models (Figures S47–S53).

A structural analysis of NO-PI-3-COF and its precursors via XRPD reveals high crystallinity, represented by four narrow reflections in the diffraction pattern of NO-PI-3-COF at 2*θ* = 5.6, 9.7, 11.3, and 14.9°, indexed as 100, 110, 200, and 120 reflections (space group *P*6̅), and a broad stacking reflection 00*l* at 2*θ* = 25.8° (Figure 2a). Compared to its parent imine and amine structures, both the apparent hexagonal symmetry and crystallinity are retained during the multistep conversion, while a significant shift of the broad stacking reflection 00*l* toward larger angles appears in NO-PI-3-COF at 2*θ* = 25.8°. Notably, the individual steps of linkage modification can be traced by a characteristic shift of the broad stacking reflection. For the reduction from imine to amine linkages, a shift toward lower angles, namely, from 2*θ* = 25.7 to 25.2°, is visible.^18^ The following oxidation to the nitrone shifts this reflection in the reverse direction, to a higher angle of 2*θ* = 25.8°, hinting at a contraction of the stacking distance in NO-PI-3-COF compared to the imine and amine derivative.

**Figure 3:**
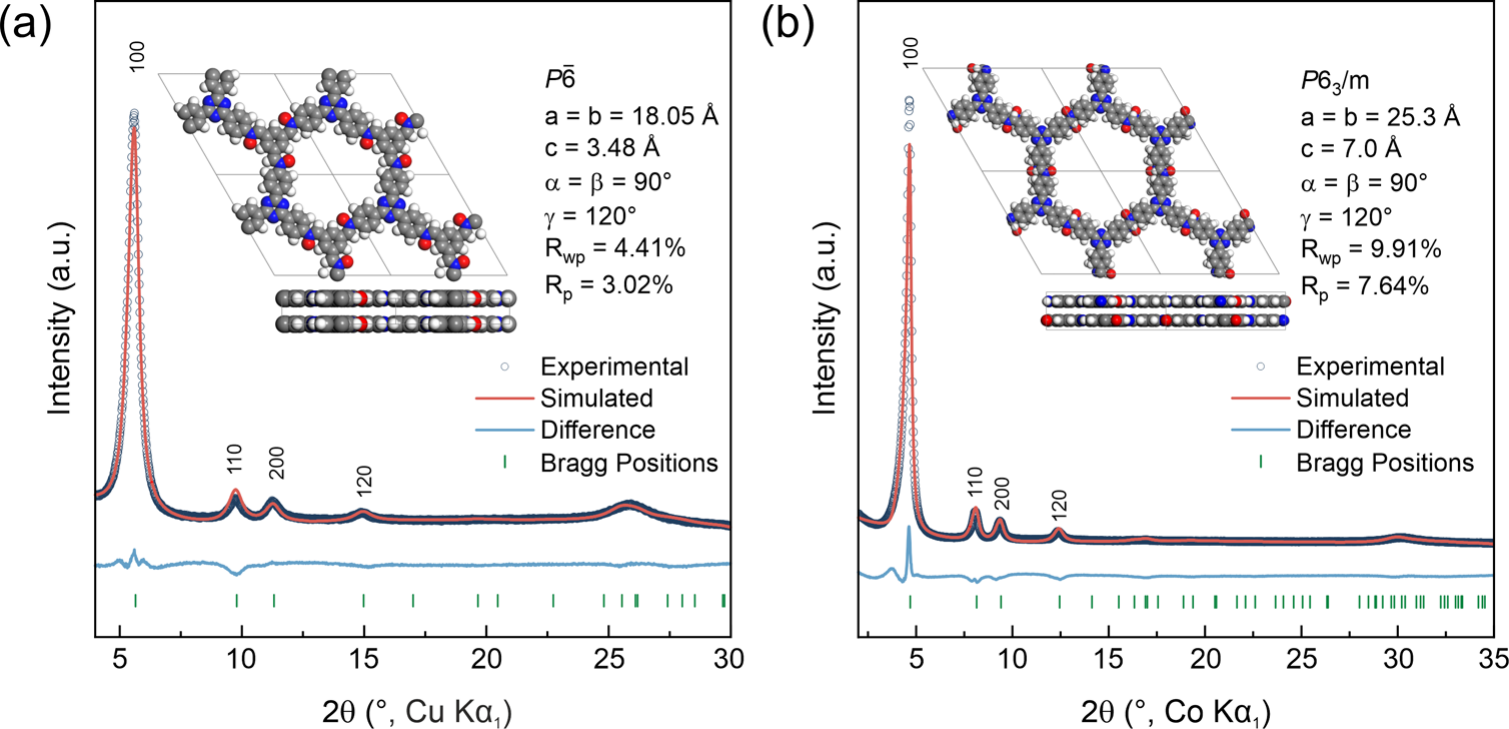
Rietveld refinements for NO-PI-3-COF (a) and NO-TTI-COF (b).

A Rietveld^20^ refinement (Figure 3) gives slightly larger in-plane unit cell parameters of *a* = *b* = 18.049(14) Å and a decreased stacking distance of *c* = 3.484(2) Å in NO-PI-3-COF (*a* = 18.034(7) Å and *c* = 3.5058(12) Å for PI-3-COF)^18^ (Table S1). While reduction to rPI-3-COF has been previously described to increase both in-plane (*a*,*b*) and stacking (*c*) cell parameters, due to increased C–N single- vs double-bond lengths and steric repulsion of the benzylic (CH_2_) protons of adjacent layers in the amine-linked material, the oxidation of amine to nitrone linkages causes the reverse effect, giving reduced in-plane and stacking cell parameters for NO-PI-3-COF. The in-plane cell parameter *a* is ∼0.02 Å larger, compared to PI-3-COF, which is in line with the steric demand of the oxygen substituent in the nitrone. The stacking distance *c* is reduced by 0.03 Å in the nitrone, suggesting a closer packing of the layers in the *c*-direction. On the other hand, a comparison of the pore size distributions obtained from N_2_ gas adsorption experiments (Figure S19) shows a small contraction of the average pore size from 1.7 nm (PI-3-COF) to 1.6 nm in NO-PI-3-COF (Figure S20). In contrast to the increased cell parameter *a*, the slight reduction in pore diameter might be affected by increasing stacking disorder, e.g., random offset-stacking of the layers^18,21,22^ upon oxidation to the nitrone, likely introduced by the reorganization of the local structure of the linkages affecting interlayer interactions.^23,24^ Despite these changes in the structure, the material shows porosity after the conversion of the linkage, evident from a BET surface area of 664 m^2^/g for NO-PI-3-COF (Figure S22). Drying of the as-synthesized material in a desiccator (CaCl_2_), instead of applying high vacuum at 120 °C, reduces drying-induced stress in the material and allowed us to obtain NO-PI-3-COF with a larger surface area of 1186 m^2^/g (Figure S30).

Motivated by these promising results, we further attempted a direct conversion of the imine linkages in PI-3-COF to nitrones with mCPBA under similar conditions (see the Supporting Information). As reported for small molecular imines, oxidation with mCPBA usually leads to a product mixture, containing the oxaziridine as a major product.^25^ Depending on the substituents, the three-membered ring of the oxaziridine rearranges under light or thermal stimulation to the nitrone or amide moiety.^26,27^ In many cases, these circumstances lead to a complex mixture of these compounds in the crude reaction mixture, which need to be separated by tedious chromatographic techniques. Due to the incompatibility of a solid-state material such as a COF to chromatographic purification, a postsynthetic linkage transformation requires a selective and efficient reaction to a single product, which would clearly exclude this synthetic strategy to obtain nitrone-linked COFs. Surprisingly, treating PI-3-COF with 1.0 equiv. mCPBA (see the Supporting Information for details) led to a clean and direct conversion of the imine linkages to nitrones. This intriguing observation highlights that preorganizing molecules in the solid state, and thereby restricting their mobility and accessibility, can confine the reaction environment and lead to unexpected selectivity of reactions of or with solid-state materials.^28,29^ Likewise, the mechanism of oxidation seems to occur via direct oxygen transfer to the imine nitrogen in PI-3-COF by mCPBA, contrary to the nucleophilic Baeyer–Villiger reaction or concerted oxidation mechanisms considered to yield oxaziridines from small-molecule imines.^30^ Furthermore, we believe that the presence of an oxaziridine linkage intermediate is unlikely because the highly strained three-membered oxaziridine heterocycle would require a drastic deformation, i.e., corrugation, of the layers. Likewise, oxaziridines pointing into the interlayer space, towards the neighboring layers, would cause an expansion of the interlayer stacking distance, which is in stark contrast to the observed reduction of the stacking distance in the nitrone (Figure 2a). The absence of any aliphatic carbon signals in the ^13^C ssNMR spectrum (Figure 2b) and nitrogen signals in the ^15^N ssNMR spectrum (Figure 2c) relating to oxaziridine formation supports our hypothesis that, in the sterically crowded COF pore, oxidation likely occurs through an electrophilic attack of mCPBA on the nitrogen,^31^ as similarly observed for sterically hindered small-molecule *N*-alkyl imines.^32^ The electrophilic attack mechanism involves an equatorial advance of the mCPBA reagent toward the linkage nitrogen, i.e., from within the pores, perpendicular to the stacking direction of the layers. In contrast, the intermediate formation of oxaziridines would involve an axial approach of mCPBA, which is sterically blocked by the neighboring layers in the 2D COF and thus rather unlikely to occur.

To further demonstrate the general transferability of our oxidation method, we successfully applied it to another imine-linked COF with a larger pore diameter, namely, the TTI-COF system (Figure S3). Analogous to the oxidation of PI-3-COF, XRPD patterns of NO-TTI-COF show retention of crystalline order throughout the reduction and oxidation steps (Figure S5). As earlier reported for the reduction of TTI-COF to amine-linked rTTI-COF, changes of the apparent symmetry occur, as evident from the disappearance of peak splitting in the XRPD pattern (Figure S5).^18^ A change from antiparallel slip-stacked TTI-COF^33^ to more eclipsed-like stacking in rTTI-COF, due to a randomization of stacking offset upon reduction, is even preserved during subsequent oxidation to NO-TTI-COF. The interlayer distance of the randomly stacked rTTI-COF (3.504(2) Å)^18^ is further reduced to 3.478(25) Å in NO-TTI-COF, following similar trends as described for NO-PI-3-COF. A comparison of the N_2_ adsorption isotherms (Figure S24) reveals retention of porosity during oxidation of rTTI to NO-TTI-COF, attested by an almost unaltered BET surface area of 1325 m^2^/g for NO-TTI-COF (from 1397 m^2^/g for rTTI-COF). Similar to NO-PI-3-COF, pore size distribution analysis shows a contraction of the pore diameter by 0.2–2.1 nm in NO-TTI-COF (Figure S25).

### Water Adsorption Properties

After synthesizing NO-PI-3-COF and handling it in ambient air, we noticed a broad signal centered at ν ≈ 3350 cm^–1^ in the FT-IR spectrum (Figure S2), which disappeared after extensive drying of the material under reduced pressure. Likewise, an intense signal at δ = 4.5 ppm in the ^1^H ssNMR spectrum of NO-PI-3-COF, as well as minor intensity changes in the ^13^C ssNMR spectrum around δ = 150 and 119 ppm were visible, referring to carbons in close proximity to the nitrone center (Figures 2b and S18). Due to the characteristic vibration in the FT-IR spectrum, we attributed the signal to water in NO-PI-3-COF, which was captured from ambient air. This observation encouraged us to study the water adsorption properties of the nitrone-linked frameworks.

**Figure 4:**
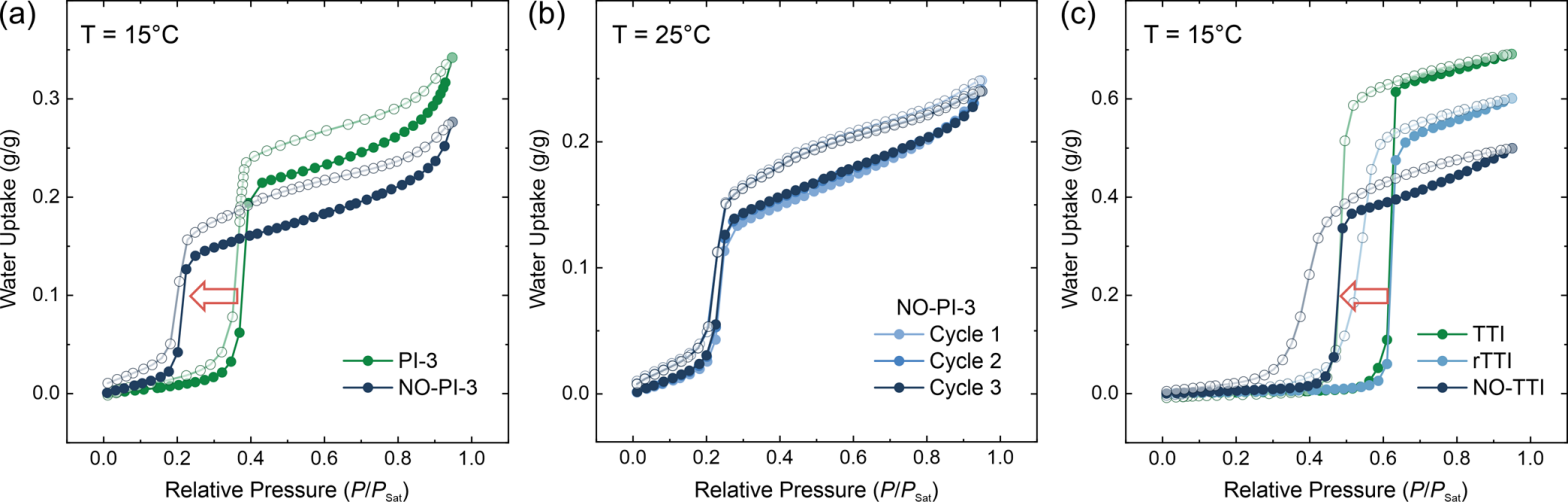
(a) Comparison of water vapor adsorption isotherms of PI-3- and NO-PI-3-COF. (b) Water adsorption–desorption cycles of NO-PI-3-COF. (c) Adsorption isotherms of TTI-, rTTI-, and NO-TTI-COF.

Water vapor adsorption experiments of NO-PI-3-COF and PI-3-COF at 15 °C (Figure 4a) show S-shaped isotherms with a steep uptake step as an effect of nucleated condensation in the pore channels.^7,34^ Relative pressures, i.e., relative humidity, at which steep pore filling occurs, shift from *P*/*P*_sat_ ≈ 0.38 (PI-3-COF) to *P*/*P*_sat_ ≈ 0.21 upon oxidation to the nitrone-linked material. The total uptake at *P*/*P*_sat_ = 0.95 is slightly reduced in NO-PI-3-COF with an adsorbed mass of 0.27 g/g of material (compared to 0.34 g/g of PI-3-COF), which correlates with the structural rearrangement as well as the increase in molecular mass of the material during oxidation. Besides a small hysteresis of the isotherm, low induction pressures are a prerequisite for efficient harvesting of atmospheric water.^9^ In contrast to a limited capacity, which is less relevant for the applicability of a material for water uptake, the induction pressure for the pore-filling step is a strongly limiting factor for harvesting water under arid conditions if the material is capable of performing multiple cycles per day.^35^ To better understand the impact of oxidation on the interaction of water with the material’s surface, we determined the heats of adsorption (*Q*_st_) for the pristine and NO-functionalized COF based on the Clausius–Clapeyron equation by measuring additional adsorption isotherms at 25 and 35 °C (Figures S34 and S36). Isotherms measured at higher temperatures show a retention of the adsorption properties observed for 15 °C, accompanied by a gradual shift of the pore-filling step to *P*/*P*_sat_ ≈ 0.26 at 35 °C. Values for *Q*_st_ calculated from the kinetically limited adsorption process for small adsorbed amounts (Figure S36) suggest a predominantly hydrophobic pore channel in NO-PI-3-COF, as similarly observed for PI-3-COF (Figure S36). In contrast, higher values for the desorption process in NO-PI-3-COF indicate a stronger interaction of water molecules with dedicated sites in the material, visible from increased *Q*_st_ toward small loadings during desorption.^15^ Likewise, the heats of adsorption reach a plateau for amounts corresponding to the pore-filling steps in both materials and stabilize at *Q*_st_ ≈ 47 kJ/mol for the imine and *Q*_st_ ≈ 50 kJ/mol for the nitrone, respectively, which are close to bulk water (*Q*_st_ = 44 kJ/mol).^15^ Together with increasing heats of adsorption toward zero loading during desorption (Figure S36), this hints at an increased interaction of water with the more polar nitrone sites in NO-PI-3-COF. Heats of adsorption at zero coverage calculated from CO_2_ adsorption isotherms at different temperatures (Figures S39 and S40) further corroborate this finding, giving increased values for NO-PI-3-COF (*Q*_st_ ≈ 30 kJ/mol) compared to PI-3-COF (*Q*_st_ ≈ 21 kJ/mol). Our observations suggest that the nitrone linkages act as hydrophilic centers, which support the coordination of water molecules. With increasing water vapor pressures, clustered water molecules at the linkage centers act as nucleation sites for water condensation in the pore channels.^15^ Consecutive volumetric adsorption/desorption cycles of NO-PI-3-COF at 25 °C (Figure 4b) do not show any signs of material degradation.

**Figure 5:**
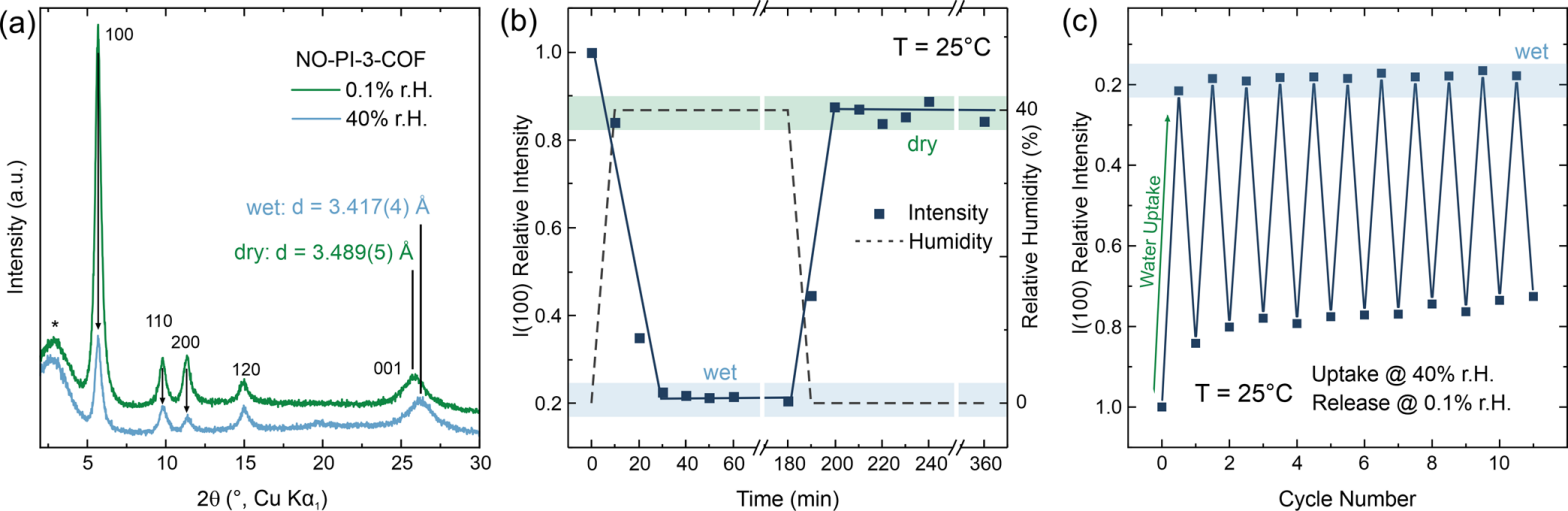
*In situ* XRPD measurements of NO-PI-3-COF (a): XRPD patterns in dehydrated (green) and hydrated states (blue) including the background diffraction signal attributed to the sample holder and the humidity chamber (asterisks) and selected reflection indices. (b) Time-dependent intensity of the 100 reflection obtained from *in situ* XRPD patterns during hydration and dehydration. (c) Modulation of the 100 intensity during multiple hydration and dehydration cycles.

In order to gain more insights into the water uptake and release behavior and cycling stability of NO-PI-3-COF, we performed *in situ* XRPD measurements at 25 °C in a dynamic atmosphere with adjustable relative humidity (Figure 5).

A gradual change of 100, 110, and 200 reflection intensity and significant upshift of the 001 reflection position occurs upon hydration (Figures 5a and S54b), which is attributed to a reduced scattering contrast and stacking distance of the layers, in agreement with simulated diffraction patterns of a refined model at different water loadings (Figure S55). Although a contraction of the interlayer distance and thus unit cell volume upon filling the pores with water molecules (Figures S54c and S56c) might appear counterintuitive, increased interlayer interactions and/or conformational changes of linker-related groups can lead to a denser packing of the COF layers. When the relative humidity is subsequently decreased, the peak intensities of 100, 110, or 200 increase again (Figures 5b and S54b), corresponding to a reversible hydration of NO-PI-3-COF analogous to the observations from volumetric water adsorption experiments (Figure 4a,b). Motivated by these results, we conjectured to use the prominent changes in 100 reflection intensity as a proxy to trace the kinetics and reversibility of the adsorption process (Figure 5b). The change in 100 intensity occurs gradually upon hydration at 25 °C (40% RH) and stabilizes after 30–40 min. Subsequent reduction of the relative humidity (0.1%) to trigger the isothermal desorption shows a similarly fast response to the signal intensity, indicating that both adsorption and desorption of water vapor occur fast enough to perform multiple cycles per day. However, the 100 intensity (i.e., the dried state) does not fully recover after the first hydration cycle, hinting at an incomplete water release under the applied isothermal desorption conditions. During consecutively performed cycles (Figure 5c), an induction period of 3–4 cycles becomes evident, after which the 100 signal intensity of the dehydrated state stabilizes. On the other hand, the corresponding relative intensity in the hydrated (i.e., wet) state stays constant at around ∼0.2 throughout the entire experiment, corroborating the observed cycling stability of the material from volumetric sorption experiments (Figure 4b) throughout an increased number of cycles.

Adsorption isotherms for NO-TTI-COF (Figure 4c) show a similar shift of the steep pore condensation step by Δ(*P*/*P*_sat_) ≈ 0.15 toward lower relative pressures. Due to the larger pore diameter of 2.1 nm, water condensation in the TTI-COF system requires higher humidity compared to PI-3-COFs, evident from the inflection point of the pore-filling step at *P*/*P*_sat_ ≈ 0.47 for NO-TTI-COF (Figure 4c). Notably, this shift is only visible after oxidation to NO-TTI-COF and does not occur after the initial reduction from TTI-COF to amine-linked rTTI-COF, highlighting the necessity of the nitrone linkage and the transferability of the observed water adsorption effect in NO-PI-3-COF to other COF systems. On the other hand, water vapor isotherms successively collected at different temperatures for TTI-, rTTI-, and NO-TTI-COF show a decrease in adsorption capacity for both postmodified TTI materials (Figure S35). In contrast to the behavior of NO-PI-3-COF, the uptake capacity of rTTI- and NO-TTI-COF is halved during the second measurement with the same material (Figure S35). XRPD (Figure S8) analysis of NO-TTI-COF after water adsorption experiments suggests that the reduction is caused by partial pore collapse, evident from a shift of the reflections towards higher angles accompanied by a loss of scattering intensity. Notably, FT-IR spectra of this material before and after water adsorption experiments do not show signs of chemical decomposition of the material such as hydrolysis (Figure S4). Solvent-induced pore collapse is a common phenomenon observed especially during the drying process of large pore COFs,^36^ which can be reduced with decreasing polarity of the solvent^37^ or by enhanced interlayer interactions.^23,38^ As previously reported for certain MOF water harvesting materials,^4^ this observation further points to a strong interaction of polar nitrone moieties in NO-TTI-COF with adsorbed water and recalls the necessity for orchestrating different types of interactions to further optimize adsorption properties of COFs. More specifically, fine-tuning of pore channel hydrophilicity can only lead to efficient water adsorption materials if interlayer interactions—as the “opponent” in 2D COFs—are likewise adjusted or, as described for NO-PI-3-COF, are strong enough to withstand drying-induced stress during water desorption.

Nevertheless, the water adsorption characteristics of NO-PI-3-COF fulfill the criteria for water harvesting materials. Besides stability under the required conditions, these materials should preferably exhibit an S-shaped water adsorption isotherm with a steep uptake step between *P*/*P*_sat_ = 0.1 and 0.3 to allow adsorption at low humidity.^9,17^ To enable energy-efficient water desorption by a small temperature swing, the isotherms should only show minor hysteresis and low heats of adsorption.^39^ Accordingly, the adsorption isotherm profile, and cycling stability of NO-PI-3-COF together with only minor hysteresis between adsorption/desorption and a *Q*_st_ close to bulk water (*Q*_st_ = 44 kJ/mol)^15^ at the pore-filling step, bodes well for the use as the active material in water harvesting applications.

## Conclusions

In summary, we present a new, facile topochemical oxidation method to obtain nitrone-linked covalent organic frameworks via solid-state synthesis starting from readily available imine-linked COFs. In contrast to earlier postsynthetic oxidation methods affording amide-linked COFs,^40,41^ our protocol makes use of the electrophilic oxidation capabilities of mCPBA^32^ and thus allows selective oxidation of nitrogen centers in the presented materials, while both crystalline order and porosity of the scaffolds are retained. Converting imine or amine linkages to nitrones introduces polar centers into the pore wall surface and thus modulates the interaction with polar adsorbates, such as water vapor. Both postsynthetically modified small and larger pore diameter nitrone COFs adsorb water vapor at reduced relative pressures compared to their parent COFs. The condensation of water vapor in the nitrone-decorated pore channels is significantly shifted by ∼20% relative humidity compared to the corresponding amine- or imine-linked precursor COFs. This makes COFs based on this novel linkage attractive candidates for atmospheric water harvesting. Due to an early onset at lower humidity, nitrone-linked COFs could be promising candidates for water vapor adsorbents in areas where arid atmospheric conditions prevail.

## Supporting Information Available

Discussions of methods and equipment used; synthetic procedures; FT-IR spectra; XRPD data and structure refinements; ^1^H, ^13^C, ^15^N ssNMR spectra; nitrogen and CO_2_ gas sorption isotherms; pore size distributions; BET plots; water vapor sorption isotherms; thermogravimetric analyses; quantum-chemically optimized structures; calculated NMR chemical shifts and *in situ* XRPD data (PDF)

## References

[ref1] Byun Y.; Je S. H.; Talapaneni S. N.; Coskun A. Advances in Porous Organic Polymers for Efficient Water Capture. Chem. – Eur. J. 2019, 25, 10262–10283. 10.1002/chem.201900940. 31022320

[ref2] Jiang S.; Meng L.; Ma W.; et al. Dual-Functional Two-Dimensional Covalent Organic Frameworks for Water Sensing and Harvesting. Mater. Chem. Front. 2021, 5, 4193–4201. 10.1039/D1QM00231G.

[ref3] Gilmanova L.; Bon V.; Shupletsov L.; et al. Chemically Stable Carbazole-Based Imine Covalent Organic Frameworks with Acidochromic Response for Humidity Control Applications. J. Am. Chem. Soc. 2021, 143, 18368–18373. 10.1021/jacs.1c07148. 34726056 PMC8587605

[ref4] Furukawa H.; Gándara F.; Zhang Y. B.; et al. Water Adsorption in Porous Metal-Organic Frameworks and Related Materials. J. Am. Chem. Soc. 2014, 136, 4369–4381. 10.1021/ja500330a. 24588307

[ref5] Stegbauer L.; Hahn M. W.; Jentys A.; et al. Tunable Water and CO_2_Sorption Properties in Isostructural Azine-Based Covalent Organic Frameworks through Polarity Engineering. Chem. Mater. 2015, 27, 7874–7881. 10.1021/acs.chemmater.5b02151.

[ref6] Biswal B. P.; Kandambeth S.; Chandra S.; et al. Pore Surface Engineering in Porous, Chemically Stable Covalent Organic Frameworks for Water Adsorption. J. Mater. Chem. A 2015, 3, 23664–23669. 10.1039/C5TA07998E.

[ref7] Nguyen H. L.; Gropp C.; Hanikel N.; et al. Hydrazine-Hydrazide-Linked Covalent Organic Frameworks for Water Harvesting. ACS Cent. Sci. 2022, 8, 926–932. 10.1021/acscentsci.2c00398. 35912353 PMC9336147

[ref8] Xu W.; Yaghi O. M. Metal-Organic Frameworks for Water Harvesting from Air, Anywhere, Anytime. ACS Cent. Sci. 2020, 6, 1348–1354. 10.1021/acscentsci.0c00678. 32875075 PMC7453559

[ref9] Kalmutzki M. J.; Diercks C. S.; Yaghi O. M. Metal-Organic Frameworks for Water Harvesting from Air. Adv. Mater. 2018, 30, 1704304 10.1002/adma.201704304. 29672950

[ref10] Lohse M. S.; Bein T. Covalent Organic Frameworks: Structures, Synthesis, and Applications. Adv. Funct. Mater. 2018, 28, 1705553 10.1002/adfm.201705553.

[ref11] Cusin L.; Peng H.; Ciesielski A.; Samorì P. Chemical Conversion and Locking of the Imine Linkage: Enhancing the Functionality of Covalent Organic Frameworks. Angew. Chem. 2021, 133, 14356–14370. 10.1002/ange.202016667. 33491860

[ref12] Helweh W.; Flanders N. C.; Wang S.; et al. Layered Structures of Assembled Imine-Linked Macrocycles and Two-Dimensional Covalent Organic Frameworks Give Rise to Prolonged Exciton Lifetimes. J. Mater. Chem. C 2022, 10, 3015–3026. 10.1039/D1TC05840A.

[ref13] Tan K. T.; Ghosh S.; Wang Z.; et al. Covalent Organic Frameworks. Nat. Rev. Methods Primers 2023, 3, 1 10.1038/s43586-022-00181-z.

[ref14] Feng J.; Zhang Y. J.; Ma S. H.; et al. Fused-Ring-Linked Covalent Organic Frameworks. J. Am. Chem. Soc. 2022, 144, 6594–6603. 10.1021/jacs.2c02173. 35380432

[ref15] Tan K. T.; Tao S.; Huang N.; Jiang D. Water Cluster in Hydrophobic Crystalline Porous Covalent Organic Frameworks. Nat. Commun. 2021, 12, 6747 10.1038/s41467-021-27128-4. 34799574 PMC8604923

[ref16] Karak S.; Kandambeth S.; Biswal B. P.; et al. Constructing Ultraporous Covalent Organic Frameworks in Seconds via an Organic Terracotta Process. J. Am. Chem. Soc. 2017, 139, 1856–1862. 10.1021/jacs.6b08815. 28106987

[ref17] Nguyen H. L.; Hanikel N.; Lyle S. J.; et al. A Porous Covalent Organic Framework with Voided Square Grid Topology for Atmospheric Water Harvesting. J. Am. Chem. Soc. 2020, 142, 2218–2221. 10.1021/jacs.9b13094. 31944678

[ref18] Grunenberg L.; Savasci G.; Terban M. W.; et al. Amine-Linked Covalent Organic Frameworks as a Platform for Postsynthetic Structure Interconversion and Pore-Wall Modification. J. Am. Chem. Soc. 2021, 143, 3430–3438. 10.1021/jacs.0c12249. 33626275 PMC7953377

[ref19] Shindo H.; Umezawa B. Infrared Absorption Spectra of Aldonitrones. I. Infrared Spectra of Benzaldehyde N-Methyl and N-Phenyl Nitrones. Chem. Pharm. Bull. 1962, 10, 492–503. 10.1248/cpb.10.492. 13912021

[ref20] Rietveld H. M. A Profile Refinement Method for Nuclear and Magnetic Structures. J. Appl. Crystallogr. 1969, 2, 65–71. 10.1107/S0021889869006558.

[ref21] Pütz A. M.; Terban M. W.; Bette S.; et al. Total Scattering Reveals the Hidden Stacking Disorder in a 2D Covalent Organic Framework. Chem. Sci. 2020, 11, 12647–12654. 10.1039/D0SC03048A. 34094458 PMC8163241

[ref22] Stähler C.; Grunenberg L.; Terban M. W.; et al. Light-Driven Molecular Motors Embedded in Covalent Organic Frameworks. Chem. Sci. 2022, 13, 8253–8264. 10.1039/D2SC02282F. 35919721 PMC9297439

[ref23] Emmerling S. T.; Schuldt R.; Bette S.; et al. Interlayer Interactions as Design Tool for Large-Pore COFs. J. Am. Chem. Soc. 2021, 143, 15711–15722. 10.1021/jacs.1c06518. 34495671 PMC8485322

[ref24] Song X.; Wang Y.; Wang C.; et al. Design Rules of Hydrogen-Bonded Organic Frameworks with High Chemical and Thermal Stabilities. J. Am. Chem. Soc. 2022, 144, 10663–10687. 10.1021/jacs.2c02598. 35675383

[ref25] Krimm H. Über Isonitrone. Chem. Ber. 1958, 91, 1057–1068. 10.1002/cber.19580910532.

[ref26] Williamson K. S.; Michaelis D. J.; Yoon T. P. Advances in the Chemistry of Oxaziridines. Chem. Rev. 2014, 114, 8016–8036. 10.1021/cr400611n. 24754443 PMC4150611

[ref27] Duhamel P.; Bénard D.; Plaquevent J.-C. Isomérisation Oxaziridine-Amide Sur Gel de Silice Obtention Non Classique D’Une Liaison Peptidique. Tetrahedron Lett. 1985, 26, 6065–6066. 10.1016/S0040-4039(00)95126-8.

[ref28] Grunenberg L.; Lotsch B. V. Escaping the Horns of the COF Dilemma. Matter 2022, 5, 2482–2484. 10.1016/j.matt.2022.06.026.

[ref29] Zhang W.; Chen L.; Dai S.; et al. Reconstructed Covalent Organic Frameworks. Nature 2022, 604, 72–79. 10.1038/s41586-022-04443-4. 35388196 PMC8986529

[ref30] Kraïem J.; Ben Othman R.; Ben Hassine B. Synthesis of Oxaziridines by Oxidation of Imines with the Trichloroacetonitrile–Hydrogen Peroxide System. C. R. Chim. 2004, 7, 1119–1126. 10.1016/j.crci.2003.12.002.

[ref31] Ogata Y.; Sawaki Y. Peracid Oxidation of Imines. Kinetics and Mechanism of Competitive Formation of Nitrones and Oxaziranes from Cyclic and Acyclic Imines. J. Am. Chem. Soc. 1973, 95, 4692–4698. 10.1021/ja00795a037.

[ref32] Boyd D. R.; Coulter P. B.; McGuckin M. R.; et al. Imines and Derivatives. Part 24. Nitrone Synthesis by Imine Oxidation Using either a Peroxyacid or Dimethyldioxirane. J. Chem. Soc., Perkin Trans. 1 1990, 301–306. 10.1039/p19900000301.

[ref33] Haase F.; Gottschling K.; Stegbauer L.; et al. Tuning the Stacking Behaviour of a 2D Covalent Organic Framework Through Non-Covalent Interactions. Mater. Chem. Front. 2017, 1, 1354–1361. 10.1039/C6QM00378H.

[ref34] Hanikel N.; Pei X.; Chheda S.; et al. Evolution of Water Structures in Metal-Organic Frameworks for Improved Atmospheric Water Harvesting. Science 2021, 374, 454–459. 10.1126/science.abj0890. 34672755

[ref35] Hanikel N.; Prévot M. S.; Fathieh F.; et al. Rapid Cycling and Exceptional Yield in a Metal-Organic Framework Water Harvester. ACS Cent. Sci. 2019, 5, 1699–1706. 10.1021/acscentsci.9b00745. 31660438 PMC6813556

[ref36] Mu Z.; Zhu Y.; Li B.; et al. Covalent Organic Frameworks with Record Pore Apertures. J. Am. Chem. Soc. 2022, 144, 5145–5154. 10.1021/jacs.2c00584. 35258975

[ref37] Zhu D.; Yan Q.; Zhu Y.; et al. Solvent-Induced Incremental Pore Collapse in Two-Dimensional Covalent Organic Frameworks. ACS Mater. Lett. 2022, 4, 2368–2374. 10.1021/acsmaterialslett.2c00672.

[ref38] Diwakara S. D.; Ong W. S. Y.; Wijesundara Y. H.; et al. Supramolecular Reinforcement of a Large-Pore 2D Covalent Organic Framework. J. Am. Chem. Soc. 2022, 144, 2468–2473. 10.1021/jacs.1c12020. 35099968 PMC9173749

[ref39] Zhou X.; Lu H.; Zhao F.; Yu G. Atmospheric Water Harvesting: A Review of Material and Structural Designs. ACS Mater. Lett. 2020, 2, 671–684. 10.1021/acsmaterialslett.0c00130.

[ref40] Waller P. J.; Lyle S. J.; Osborn Popp T. M.; et al. Chemical Conversion of Linkages in Covalent Organic Frameworks. J. Am. Chem. Soc. 2016, 138, 15519–15522. 10.1021/jacs.6b08377. 27934009

[ref41] Zhou Z. B.; Han X. H.; Qi Q. Y.; et al. A Facile, Efficient, and General Synthetic Method to Amide-Linked Covalent Organic Frameworks. J. Am. Chem. Soc. 2022, 144, 1138–1143. 10.1021/jacs.1c12392. 35038262

