# Supplementary material for: Postsynthetic Transformation of Imine- into Nitrone-Linked Covalent Organic Frameworks for Atmospheric Water Harvesting at Decreased Humidity
Source: J Am Chem Soc. 2023 May 26;145(24):13241–8. doi: 10.1021/jacs.3c02572 (PMC10288504; doi:10.1021/jacs.3c02572)
Supplement: Supplementary file 1 — ja3c02572_si_001.pdf [file ja3c02572_si_001.pdf]

Supporting Information:

Postsynthetic Transformation of Imine- into Nitrone-Linked Covalent Organic Frameworks for Atmospheric Water Harvesting at Decreased Humidity

Lars Grunenberg,[a,b] Gökcen Savasci,[a,b,c] Sebastian T. Emmerling,[a,b] Fabian Heck,[a,b] Sebastian Bette,[a] Afonso Cima Bergesch,[a,b] Christian Ochsenfeld,[a,b,c] and Bettina V. Lotsch.[a,b,c,*]

[a] Max Planck Institute for Solid State Research, Heisenbergstr. 1, 70569 Stuttgart, Germany

[b] Department of Chemistry, Ludwig-Maximilians-Universität (LMU), Butenandtstr. 5-13, 81377 Munich, Germany

[c] E-conversion, Lichtenbergstr. 4a, 85748 Garching, Germany

[*] Email: b.lotsch@fkf.mpg.de

Table of Contents

Methods and Equipment 3
Synthetic Procedures 4
COF Synthesis 4
Analytical Data 6
FT-IR Spectra 6
XRPD Data and Structure Refinement 9
ssNMR Spectra 12
Nitrogen Gas Sorption Experiments 16
Water Vapor Sorption Experiments 23
CO_2 Gas Sorption Experiments 27
Thermogravimetric Analysis.. 28
Quantum Chemical Calculations 31
In situ X-ray powder diffraction:.. 33
References 36

Methods and Equipment

General methods: All reactions, unless otherwise noted, were performed with magnetic stirring under inert gas (N_2 or Ar) atmosphere using standard Schlenk techniques. Reaction temperatures were electronically monitored as external heating block temperatures. Unless otherwise noted, reagents were purchased from different commercial sources and used without further purification. Commercial *m*-CPBA was purified according to a procedure described below.

Infrared spectroscopy: IR spectra were recorded on a Perkin Elmer UATR Two FT-IR spectrometer equipped with an attenuated total reflection (ATR) measuring unit. IR data are reported in wavenumbers (cm^{-1}) of normalized absorption. The IR bands are characterized as w (weak), m (medium), s (strong), or br (broad).

Gas sorption measurements: Sorption measurements for COFs were performed on a Quantachrome Instruments Autosorb iQ MP with nitrogen at 77 K or CO_2 at designated temperature. The samples were degassed for 12 h at 120 °C under vacuum prior to the gas adsorption studies. Pore size distributions were determined from nitrogen adsorption isotherms using the QSDFT cylindrical pores in carbon model for nitrogen at 77 K. For multipoint BET surface area calculations, pressure ranges were chosen with the help of the BET assistant in the ASiQwin software, which chooses BET tags in accordance with the ISO recommendations equal or below the maximum in grams per square meter.

Values of the adsorbed amount of CO_2 in V_{STP} [cm^3g^{-1}] were converted to molar amount adsorbed per gram of material [$mmol^1g^{-1}$] = $V_{STP}/22.414$. Heats of adsorption at zero coverage (θ) were estimated from CO_2 adsorption isotherms measured at 273 K, 288 K and 298 K using Henry's law (Eq. 1). The low pressure region ($0 < p < 50$ Torr) of the isotherms was fitted linearly to derive the Henry coefficient (k_H) normalized to a proportionality factor (α) from the slope ($=k_H\alpha^{-1}$) of the fit, according to Eq. 2. The normalized Henry coefficients at respective temperatures (T) were then plotted semi logarithmically vs. T^{-1}. Heats of adsorption (ΔQ_{ST}) were then calculated from the slope of the linear fits (Eq. 3).

$$\theta = \propto V_{STP} = \frac{k_H p}{1+k_H p} \approx k_H p \text{ (for small pressures)} \quad (1)$$

$$V_{STP} \approx \frac{k_H}{\propto} p \quad (2)$$

$$\ln\left(\frac{k_H}{\propto}\right) = -\frac{\Delta Q_{ST}}{R} \cdot \frac{1}{T} - \ln(\propto) \quad (3)$$

Vapor sorption measurements: Vapor sorption experiments were performed on a Quantachrome Instruments Autosorb iQ MP with water at different temperatures. The samples were degassed for 12 h at 120 °C under vacuum prior adsorption studies. Values of the adsorbed amount V_{STP} [cm^3g^{-1}] were converted to gravimetric amount adsorbed per gram of material [g^1g^{-1}] = $V_{STP}/22414*18.015$.

X-ray powder diffraction (XRPD): X-ray powder diffraction experiments were performed on a Stoe Stadi P diffractometer (Co-/Cu-Kα_1, Ge(111)) in Debye-Scherrer geometry. The samples were measured in sealed glass capillaries (OD = 0.7 mm) and spun for improved particle statistics.

Rietveld refinements: Rietveld refinements were performed using TOPAS v6. The background was corrected with Chebychev polynomials (Order 5). Simple axial and zero-error corrections were used together with additional corrections for Lorentzian crystallite size and/or strain broadening.

Supercritical CO_2 activation: Activation of the methanol-soaked COF samples with supercritical CO_2 was performed on a Leica EM CPD300 critical point dryer.

Thermogravimetric analysis: Thermogravimetric analysis (TGA) was performed on a NETZSCH STA 449 F3 Jupiter. Measurements were carried out with 3-6 mg of sample in an Al_2O_3 crucible under Ar flow (60 mL/min) a temperature range between 30 and 800 °C and a heating rate of 5 K/min. Deviating buoyancy effects between the reference crucible and the sample-loaded crucible were compensated by a correction of y-offsets. Baseline correction was achieved by subtracting reference measurements with an empty crucible.

Quantum-chemical Calculations: Atom positions and lattices of all periodic structures were optimized on RI-PBE-D3/def2-TZVP[1, 2, 3, 4] level of theory using an acceleration scheme based on the resolution of the identity (RI) technique and the continuous fast multipole method (CFMM)[5, 6, 7] implemented[8, 9] in Turbomole version V7.3.[10]

The CFMM uses multipole moments of maximum order 20, together with a well-separateness value of 3 and a basis function extent threshold of 10^{-9} a.u. Grid 7 was used for the numerical integration of the exchange-correlation term. The norm of the gradient was converged to 10^{-4} a.u. and the total energy to 10^{-8} Hartree within the structure optimization using the gamma point approximation.

Structures for all investigated molecular compounds were optimized on PBE0-D3/def2-TZVP[2, 3, 11, 12] level of theory. Subsequent frequency calculations were performed on the same level of theory to ensure all minima to be true minima on the potential energy hypersurface.

NMR chemical shifts were obtained on B97-2/pcS-2[13, 14] level of theory using the FermiONs++ program package.[13, 15, 16]

Purification of commercial *m*CPBA: *meta*-chloroperbenzoic acid (*m*CPBA) was obtained from *Merck KGaA, Darmstadt.* Commercial grade *m*CPBA contains water and impurities of benzoic acid, which were removed following a previously reported procedure.[17] *m*CPBA (25 g) was dissolved in Et_2O (200 mL) and washed with an aqueous buffer solution (pH 7, 3 x 100 mL). The organic phase was dried ($MgSO_4$). The solvent was removed under reduced pressure to afford pure *m*CPBA as white crystals. To avoid decomposition, the purified material was stored at -30°C under inert atmosphere in the dark.

Synthetic Procedures

COF Synthesis

Synthesis of PI-3-COF: PI-3-COF was synthesized according to a previously described procedure.[18] To a mixture of benzene-1,3,5-tricarbaldehyde (21.4 mg, 0.13 mmol, 1.0 equiv.) and 4,4',4''-(1,3,5-triazine-2,4,6-triyl)trianiline (46.8 mg, 0.13 mmol, 1.0 equiv.) in mesitylene (2.7 mL) and 1,4-dioxane (1.3 mL), aqueous 6 M AcOH (0.5 mL) was added. The suspension was heated at 120°C for 72 h. The precipitate was collected via suction filtration, washed with DMF (20 mL), THF (20 mL) and DCM (20 mL) and extracted with MeOH in a Soxhlet extractor for 12 h. Activation with supercritical CO_2 afforded PI-3-COF (55.0 mg, 90%) as a yellow solid.

Synthesis of rPI-3-COF: rPI-3-COF was synthesized according to a previously described procedure.[18] To a suspension of PI-3-COF (30.0 mg) in mesitylene (2 mL) and 1,4-dioxane (1 mL), formic acid (97%, 53.0 µL) was added. The suspension was heated at 120°C for 48 h. The precipitate was collected via suction filtration and extracted with MeOH in a Soxhlet extractor for 12 h. Activation with supercritical CO_2 afforded rPI-3-COF (28.0 mg, 92%) as a yellow solid.

Synthesis of NO-PI-3-COF: (a) from rPI-3-COF: To a cooled suspension of rPI-3-COF (10.4 mg, 22.2 µmol, 1.0 equiv.) in DCM (1.0 mL), a solution of mCPBA (21.1 mg, 134 µmol, 6.0 equiv.) in DCM (3.0 mL) was added at 0°C. The reaction mixture was stirred for 24 h at room temperature. The solid was filtered and washed with acetone and methanol (3 x 10 mL each). Soxhlet extraction with methanol, supercritical drying (CO_2) and drying under high vacuum afforded NO-PI-3-COF (8.40 mg, 75%) as a yellow-orange solid.

(b) from PI-3-COF: To a cooled suspension of PI-3-COF (30.0 mg, 64.9 µmol, 1.0 equiv.) in DCM (4.0 mL), a solution of mCPBA (33.9 mg, 195 µmol, 3.0 equiv.) in DCM (2.0 mL) was added dropwise at 0°C. The reaction mixture was stirred for 18 h, during which the mixture was allowed to warm to room temperature. The crude product was filtered and washed with acetone (3 x 10 mL) and methanol (3 x 10 mL). Soxhlet extraction with methanol for 24 h, supercritical drying with CO_2 and drying under high vacuum afforded NO-PI-3-COF (27.1 mg, 82%) as a yellow-orange powder. Notably, drying of the material in a desiccator ($CaCl_2$) allowed to obtain NO-PI-3-COF with higher porosity (Figure S 30).

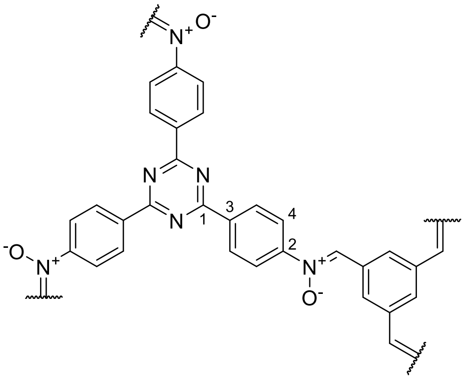

^{1}H ssNMR (MAS, 400 MHz): δ = 7.7 (C-4, C-Ar) ppm.

^{13}C ssNMR (CP-MAS, 101 MHz): δ = 169.2 (C-1), 149.7 (C-2), 136.3 (C-3), 129.5 (C-Ar), 121.2 (C-Ar), 117.3 (C-4) ppm.

^{15}N ssNMR (CP-MAS, 61 MHz): δ = −106.3 (N-2), −129.5 (N-1) ppm.

FT-IR (ATR): ν = 1596 (w), 1514 (s), 1413 (w), 1367 (m), 1180 (w), 1145 (w), 1013 (w), 867 (w), 812 (m), 676 (w), 524 (w) cm^{-1}.

Synthesis of TTI-COF: TTI-COF was synthesized according to a previously described procedure.[18] A mixture of 4,4',4''-(1,3,5-triazine-2,4,6-triyl)tribenzaldehyde (25.0 mg, 63.5 µmol, 1.0 equiv.) and 4,4',4''-(1,3,5-triazine-2,4,6-triyl)trianiline (22.5 mg, 63.5 µmol, 1.0 equiv.) in mesitylene/1,4-dioxane 1:1 (5 mL) and aqueous 6 M AcOH (0.13 mL) was heated at 120°C for 72 h. The precipitate was collected via suction filtration, washed with DMF (20 mL), THF (20 mL) and DCM (20 mL) and extracted with MeOH in a Soxhlet extractor for 12 h. Activation with supercritical CO_2 afforded TTI-COF (26.4 mg, 60%) as a yellow solid.

Synthesis of rTTI-COF: rTTI-COF was synthesized according to a previously described procedure.[18] To a suspension of TTI-COF (100.0 mg) in mesitylene (6 mL) and 1,4-dioxane (6 mL), formic acid (97%, 64.9 µL) was added. The suspension was heated at 120°C for 48 h. The precipitate was collected via suction filtration and extracted with MeOH in a Soxhlet extractor for 12 h. Activation with supercritical CO_2 afforded rTTI-COF (97.0 mg, 96%) as a yellow solid.

Synthesis of NO-TTI-COF: To a cooled suspension of rTTI-COF (30.0 mg, 42.9 µmol, 1.0 equiv.) in acetone (3.0 mL), a solution of mCPBA (45.6 mg, 0.26 mmol, 6.0 equiv.) in acetone (2.0 mL) was added dropwise at 0°C. The reaction mixture was stirred for 24 h, during which the mixture was allowed to warm to room temperature. The crude product was filtered and washed with acetone (3 x 10 mL) and methanol (3 x 10 mL). Soxhlet extraction with methanol for 24 h and supercritical drying with CO_2 afforded NO-TTI-COF (23.0 mg, 72%) as a yellow-orange powder.

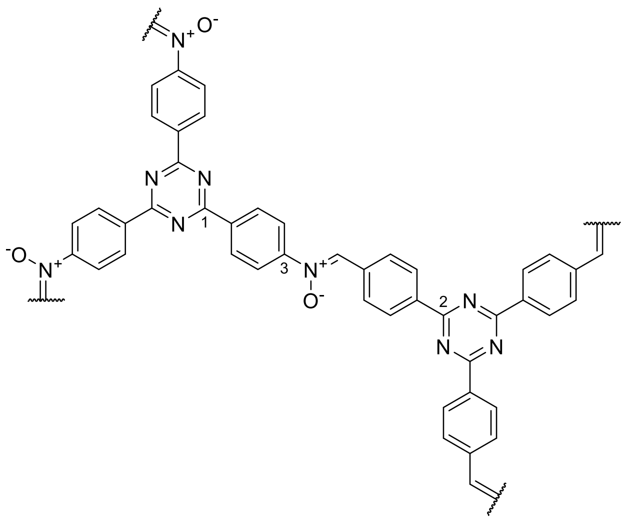

^{1}H ssNMR (MAS, 400 MHz): δ = 7.2 (C-Ar) ppm.

^{13}C ssNMR (CP-MAS, 101 MHz): δ = 168.9 (C-1, C-2), 149.6 (C-3), 136.4 (C-Ar), 128.0 (C-Ar), 119.5 (C-Ar) ppm.

^{15}N ssNMR (CP-MAS, 61 MHz): δ = −95.4 (N-3), −128.0 (N-1, N-2) ppm.

FT-IR (ATR): ν = 1596 (w), 1580 (w), 1511 (s), 1364 (m), 1315 (w), 1244 (w), 1179 (w), 1015 (w), 868 (w), 812 (m), 523 (w) cm^{-1}.

Analytical Data

FT-IR Spectra

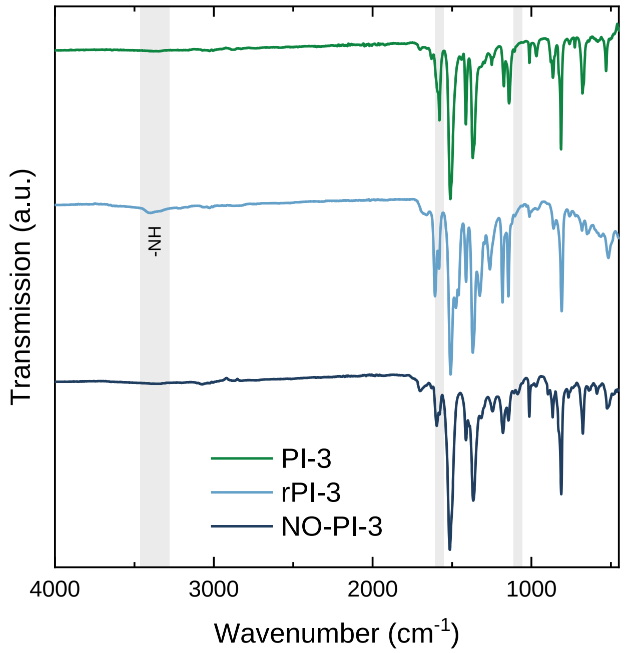

Figure S 1: FT-IR spectra comparison of NO-PI-3-COF and its parent amine-linked (rPI-3) and imine-linked (PI-3) covalent organic frameworks. Secondary amine vibrations present in rPI-3-COF vanish upon oxidation to nitrone-linkages in NO-PI3-COF. Further but less prominent changes in the finger print region (gray) differentiate NO-PI-3-COF from PI-3-COF.

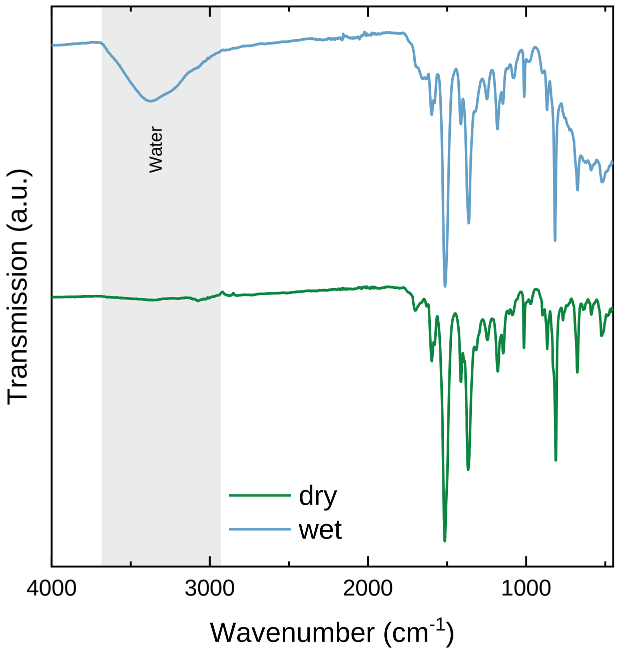

Figure S 2: FT-IR spectra comparison of NO-PI-3-COF after exposing it to air (wet) and after drying under high vacuum (dry). The broad and intense vibration centered at ~3400 cm^{-1} suggests that the framework adsorbs water vapor from the atmosphere.

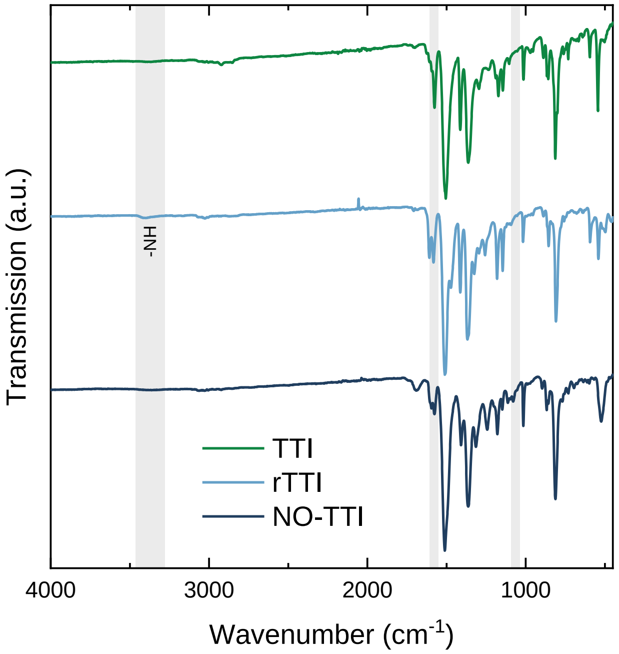

Figure S 3: FT-IR spectra comparison of NO-TTI-COF and its parent amine-linked (rTTI) and imine-linked (TTI) covalent organic frameworks. Secondary amine vibrations present in rTTI-COF vanish upon oxidation to nitrone-linkages in NO-TTI-COF. Further, but less prominent changes in the fingerprint region (gray) differentiate NO-TTI-COF from TTI-COF.

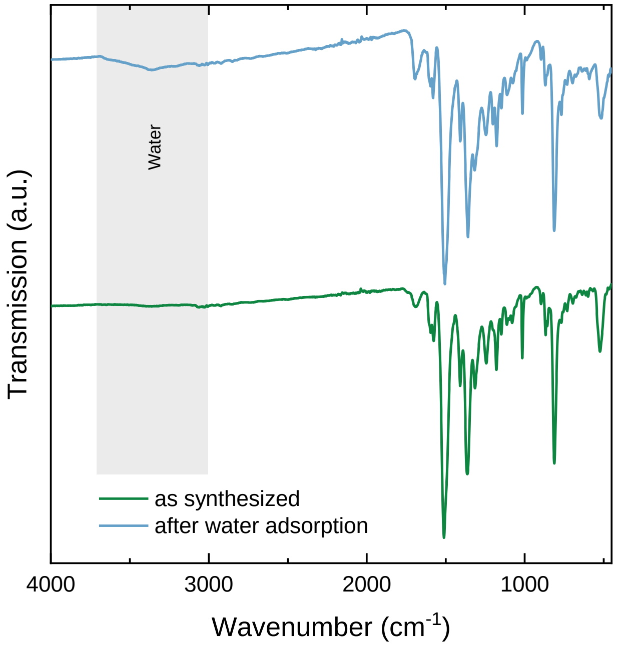

Figure S 4: FT-IR spectra comparison of NO-TTI-COF as-synthesized and after water vapor adsorption experiments. Despite the vibrations related to adsorbed water, especially the fingerprint region of the spectrum remained largely unchanged, suggesting that changes in the properties are rather related to structural changes in the material, instead of changes in the chemical connectivity.

XRPD Data and Structure Refinement

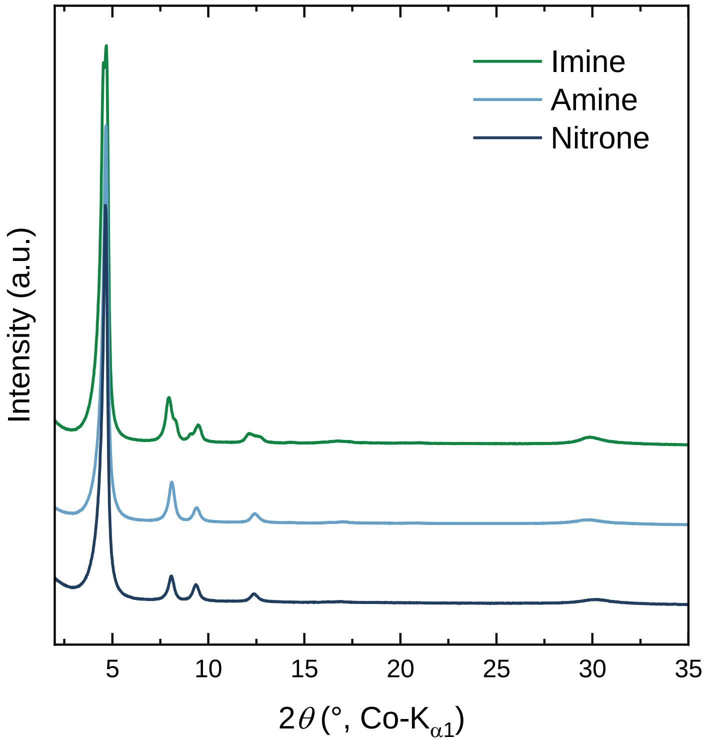

Figure S 5: XRPD comparison of TTI, rTTI and NO-TTI-COF. Peak splitting in TTI-COF occurs due to antiparallel slip-stacking of the layers. [18] *After reduction and oxidation more eclipsed-like stacking occurs, due to a randomization of stacking offset.* [18]

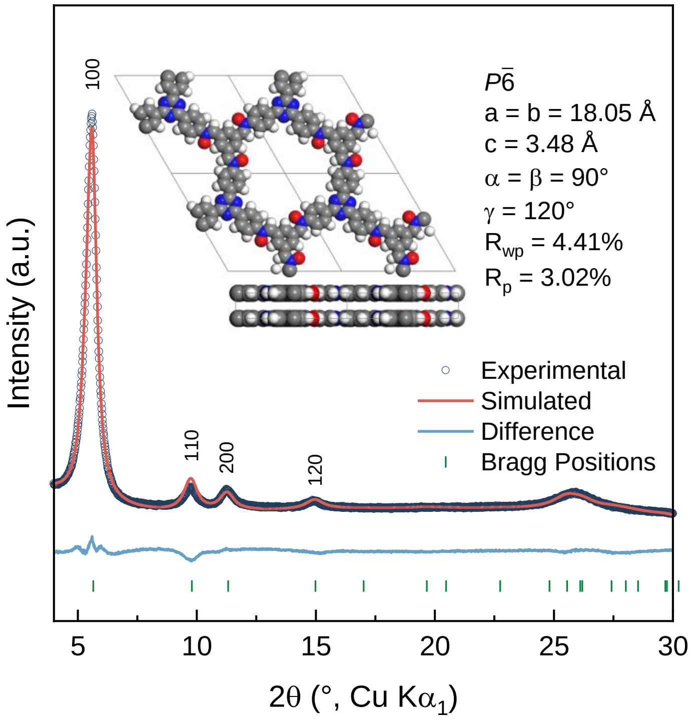

Figure S 6: Experimental XRPD pattern and Rietveld refinement for NO-PI-3-COF.

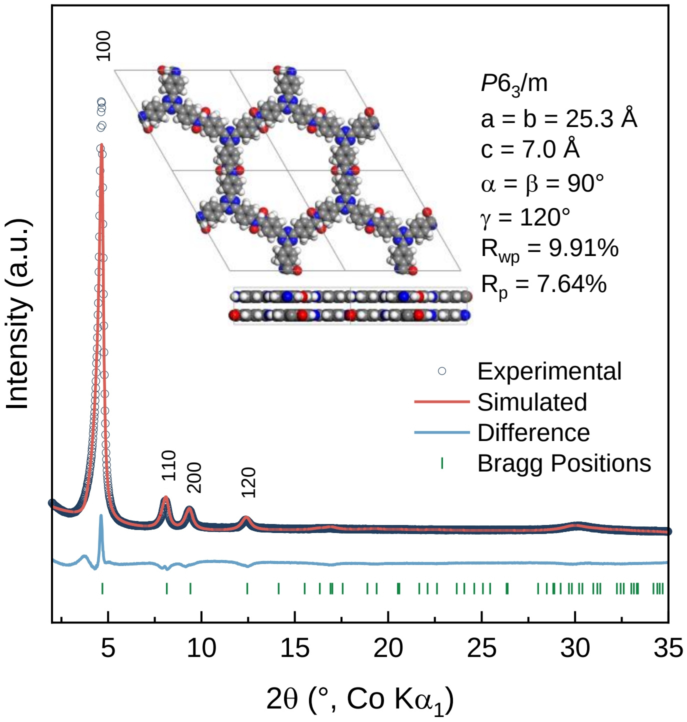

Figure S 7: Experimental XRPD pattern and Rietveld refinement for NO-TTI-COF.

Table S 1: Cell parameters of Rietveld refined nitrone-linked COFs.

**Fitted Pattern**	**NO-PI-3-COF**	**NO-TTI-COF**
**Space group**	$P\bar{6}$	$P6_3/m$
**R_{wp} (%)**	4.41	9.91
**R_p (%)**	3.02	7.64
**a (Å)**	18.049(14)	25.209(4)
**b (Å)**	18.049(14)	25.209(4)
**c (Å)**	3.484(2)	6.956(5)
**α (°)**	90	90
**β (°)**	90	90
**γ (°)**	120	120

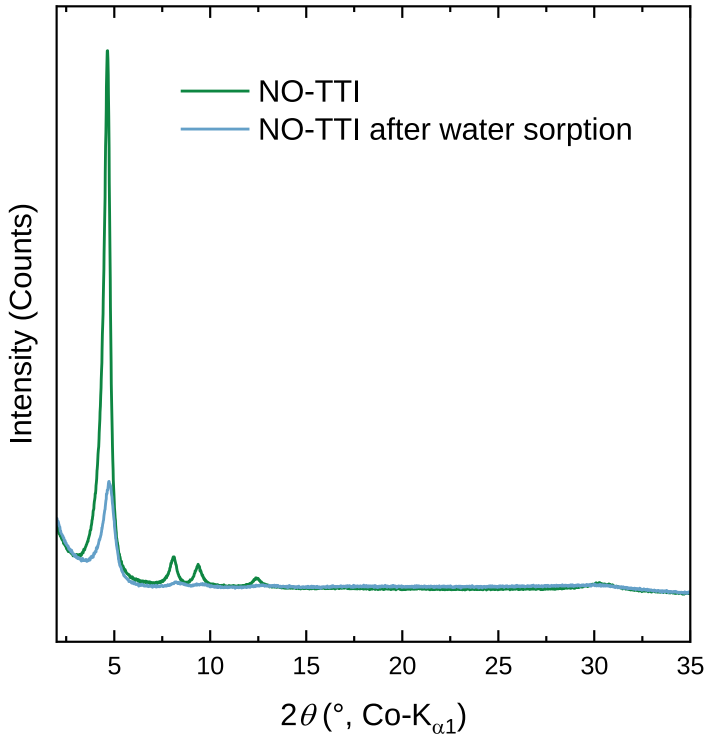

Figure S 8: XRPD patterns of NO-TTI before and after water vapor adsorption experiments (same sample). The observed decrease in uptake capacity (Figure S 35) is related to a decrease in structural definition of the sample, due to pore collapse.

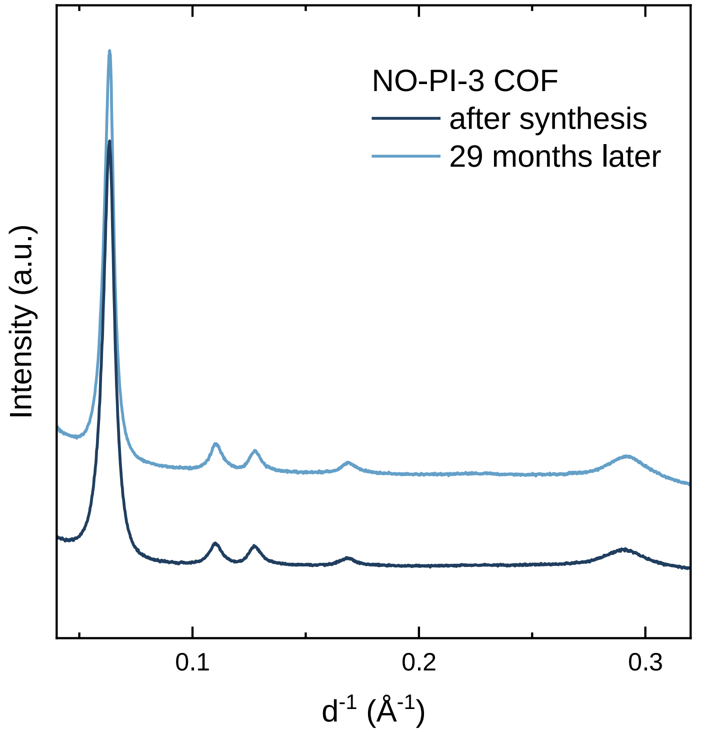

Figure S 9: XRPD patterns of NO-PI-3 COF before and after storing the sample in hydrated state under ambient conditions for 29 months.

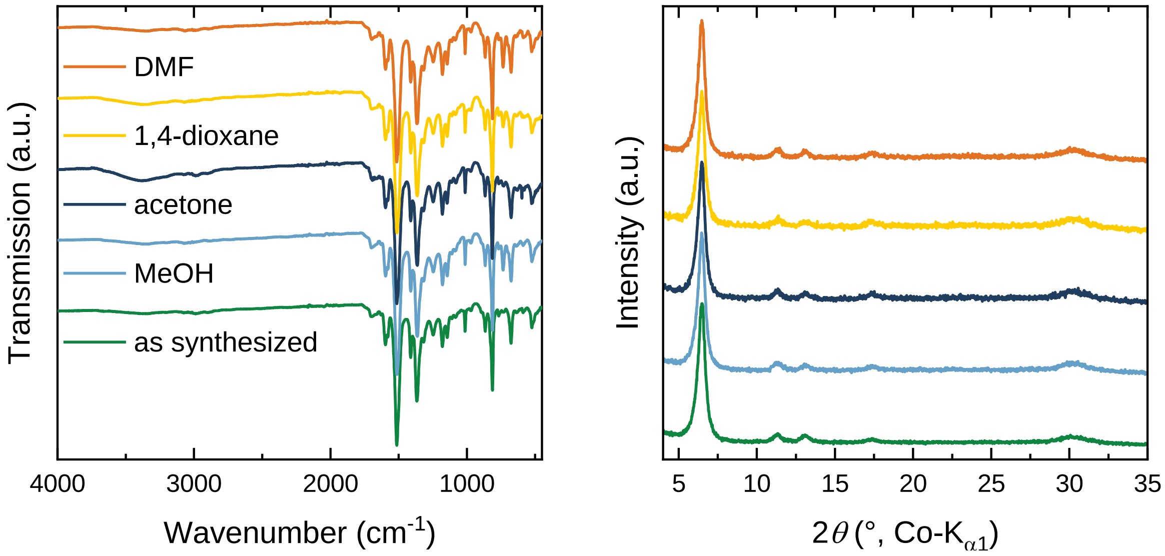

Figure S 10: FT-IR spectra (left) and XRPD patterns of NO-PI-3-COF treated with common organic solvents for 24h. The samples were subsequently extracted with DCM and dried under slowly reduced pressure. After this treatment, the samples do not show any signs of decomposition. The broad vibration at ~3400 cm^{-1} refers to adsorbed water from the atmosphere during the FT-IR experiment.

ssNMR Spectra

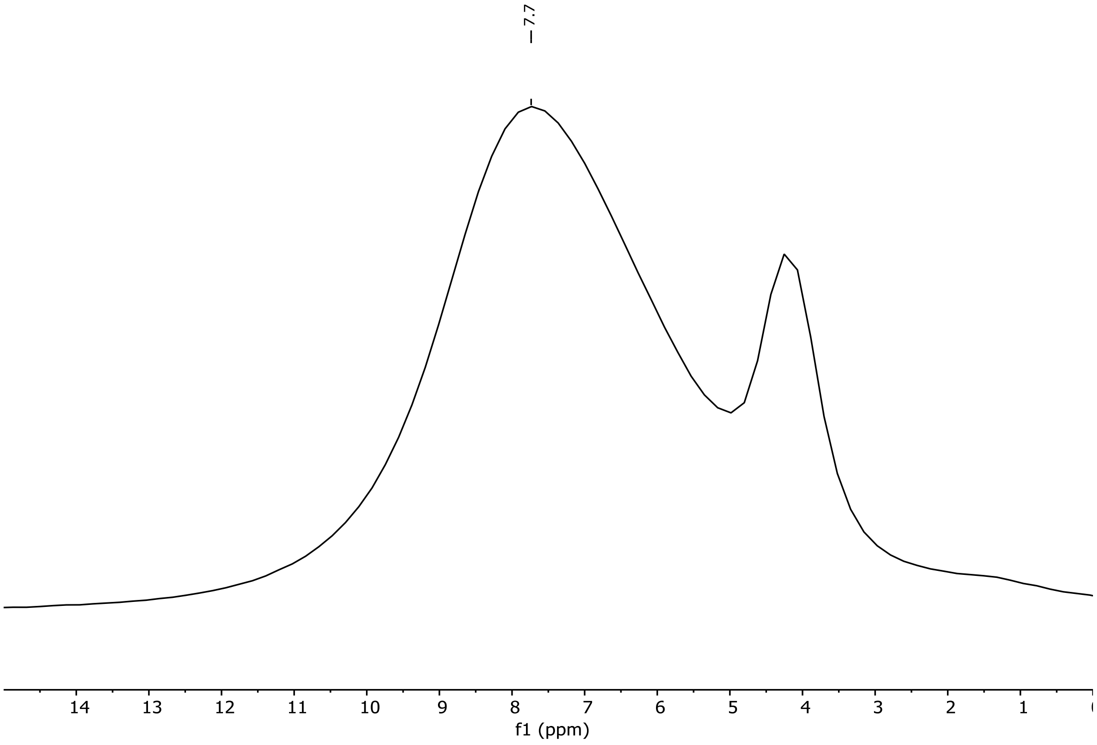

Figure S 11: ^{1}H-ssNMR spectrum of NO-PI-3-COF. Signal centered at δ ≈ 4 ppm is caused by residual adsorbed water.

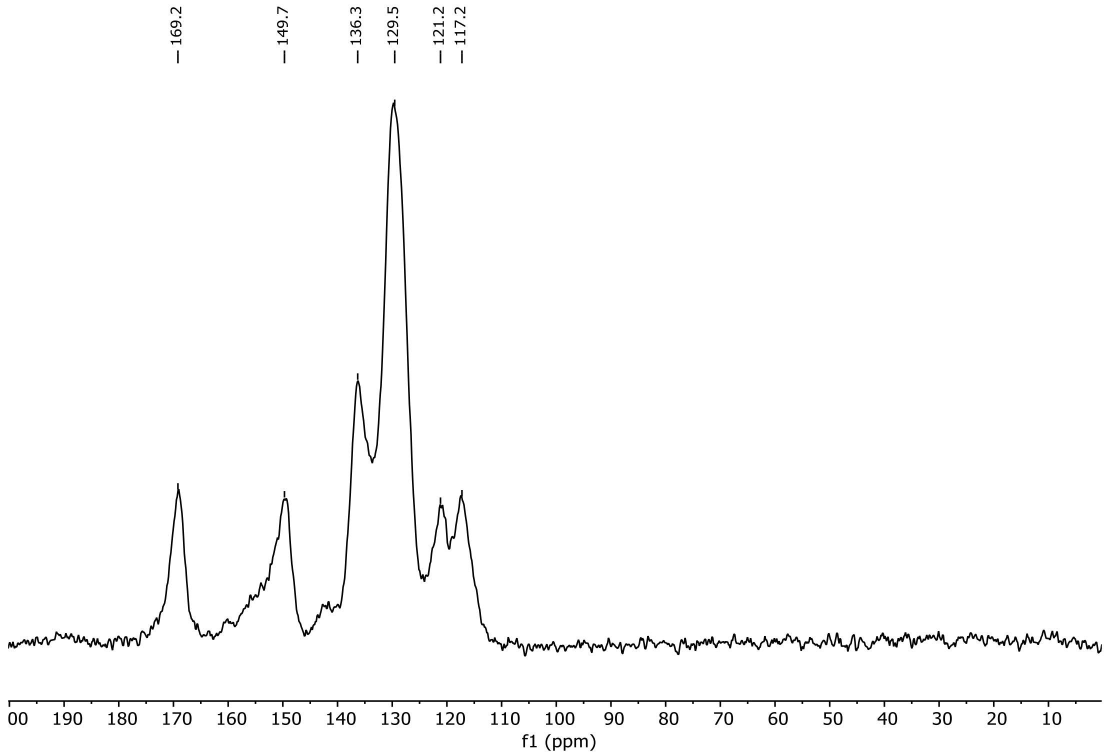

Figure S 12: ^{13}C-ssNMR spectrum of NO-PI-3-COF.

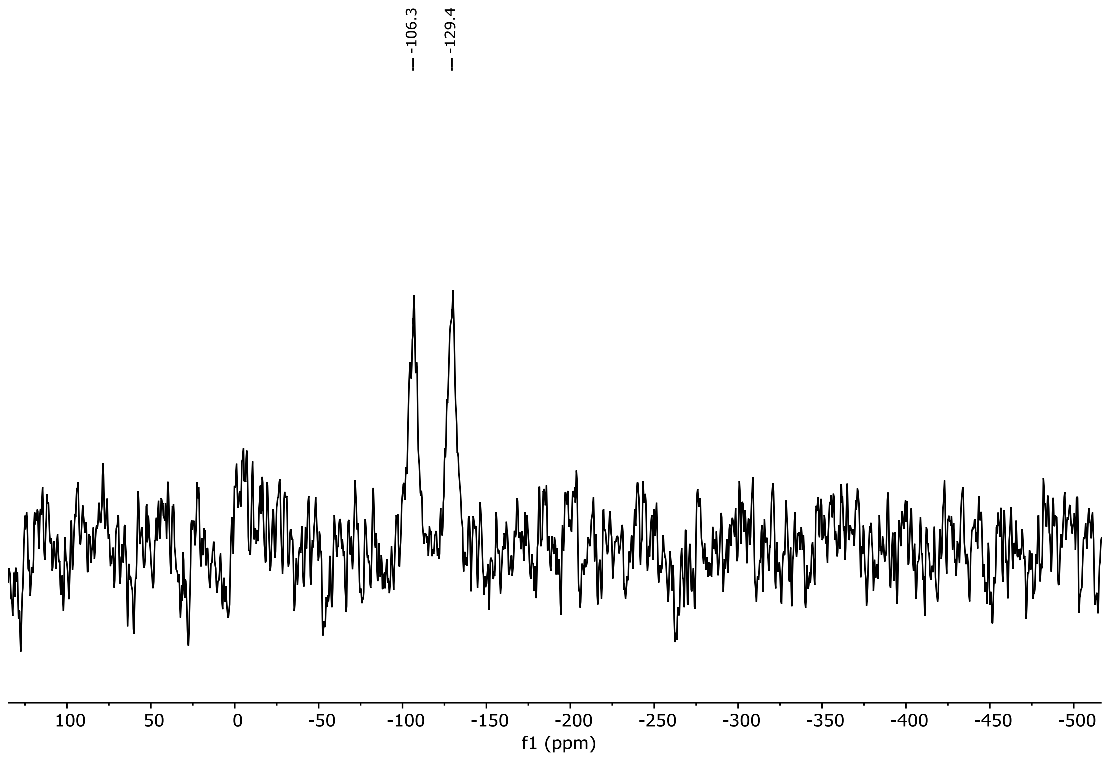

Figure S 13: ^{15}N-ssNMR spectrum of NO-PI-3-COF. The signals at δ = -129.4 ppm is assigned to the triazine-, and -106.3 ppm to the nitrone-nitrogen atoms.

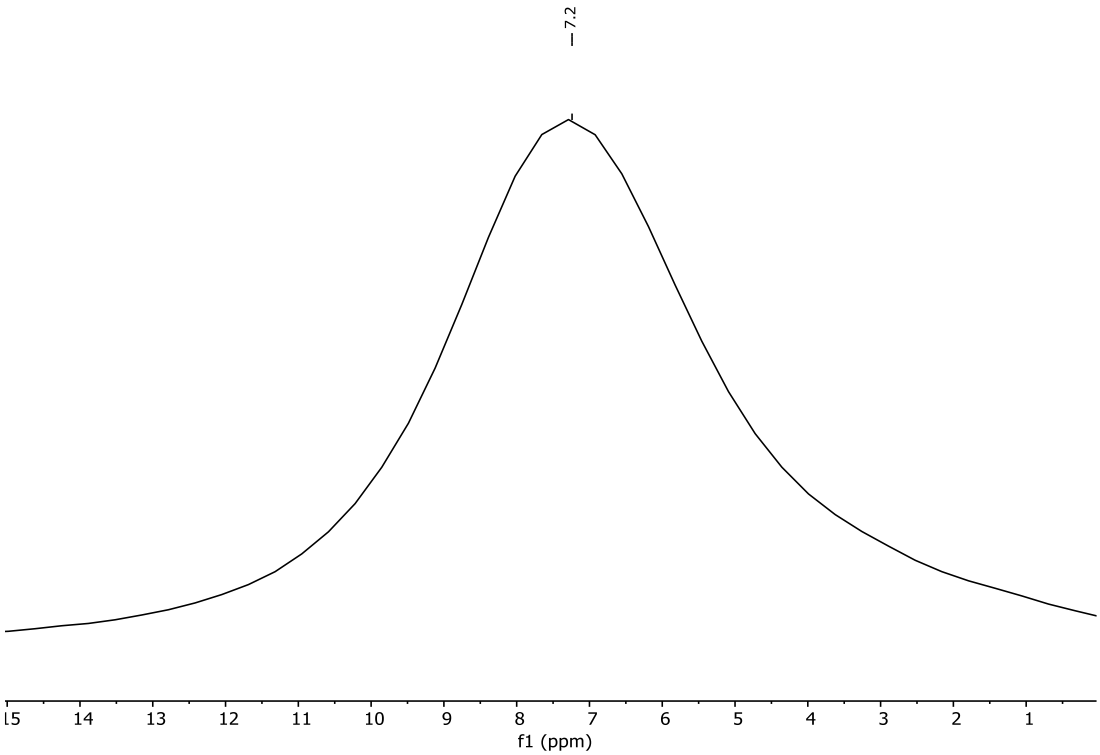

Figure S 14: ^{1}H-ssNMR spectrum of NO-TTI-COF.

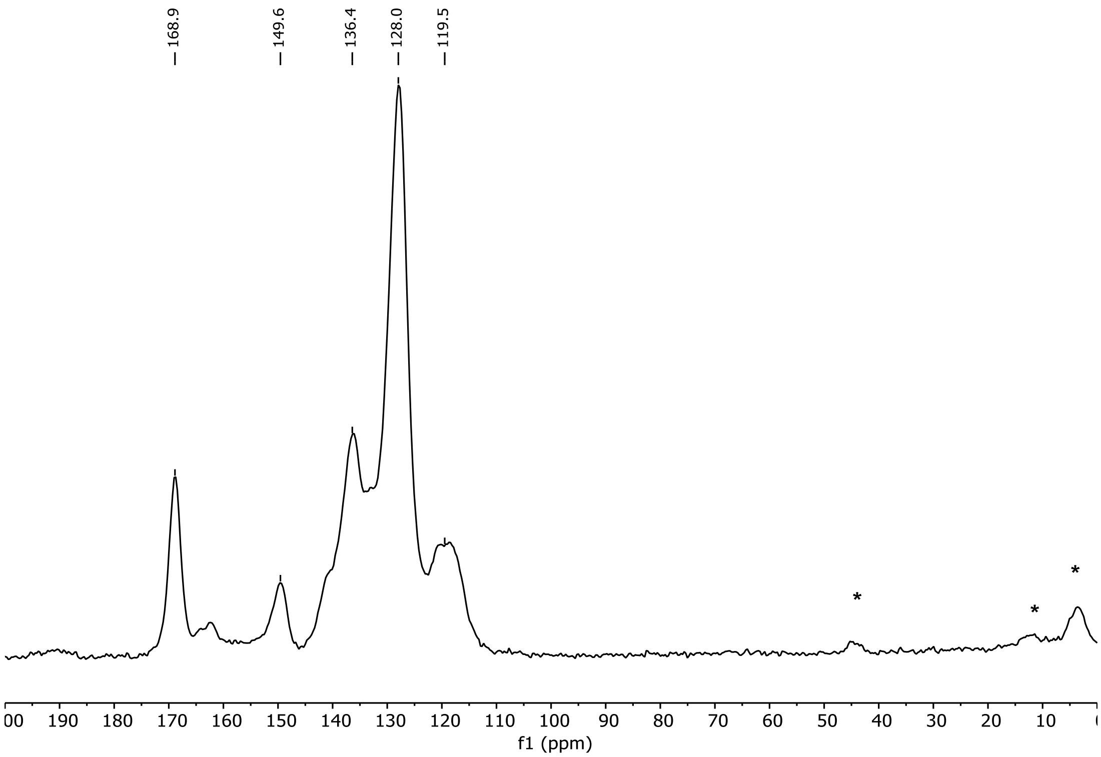

Figure S 15: ^{13}C-CP-ssNMR spectrum of NO-TTI-COF. Asterisks denote spinning sidebands.

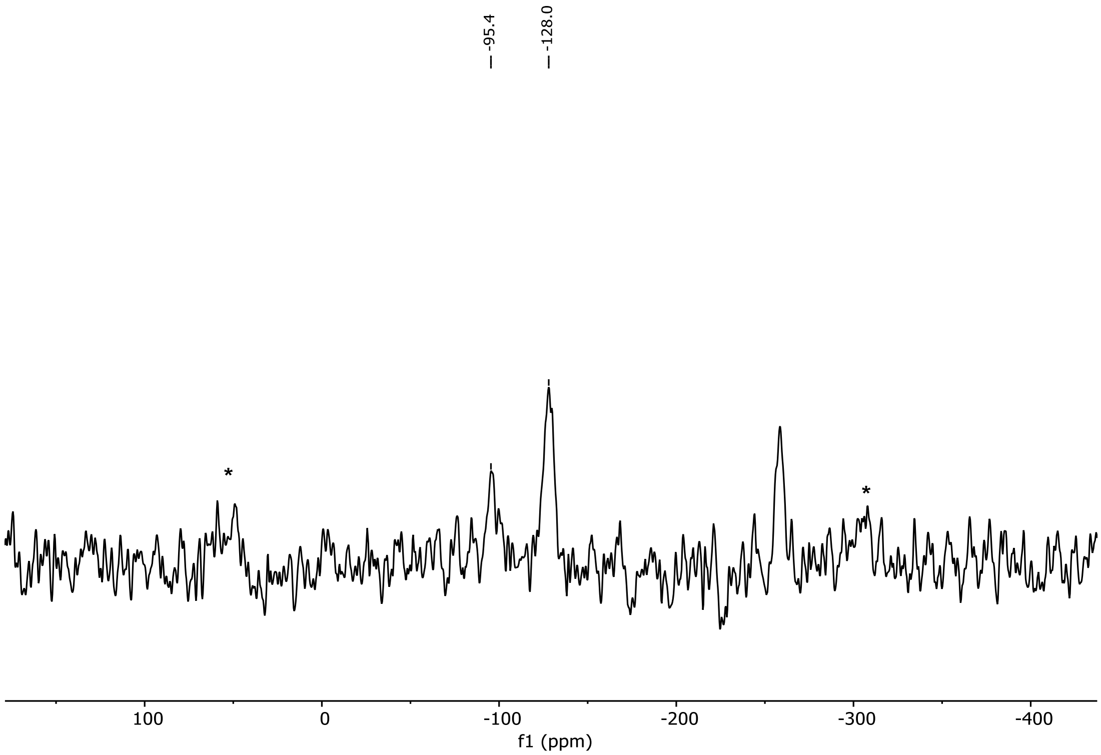

Figure S 16: ${}^{15}N$-CP-ssNMR spectrum of NO-TTI-COF. The signal at δ ≈ -260 ppm refers to traces of amide groups in the material, occurring as an impurity after oxidation. Note that their intensity is (over)amplified compared to nitrogen atoms without proton substitution, due to the N-H cross-polarization experiment. Asterisks denote spinning sidebands.

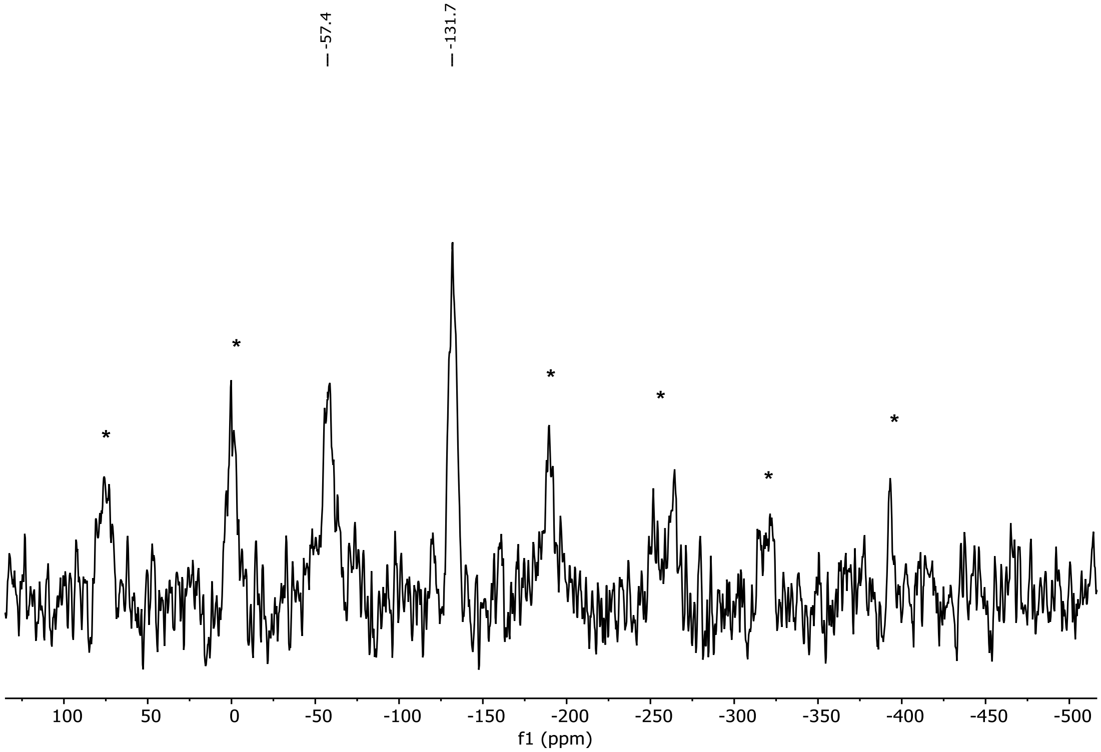

Figure S 17: ${}^{15}N$-CP-ssNMR spectrum of PI-3-COF. The signals at δ = -131.7 ppm are assigned to the triazine-, and -57.4 ppm to the imine-nitrogen atoms. Asterisks denote spinning sidebands.

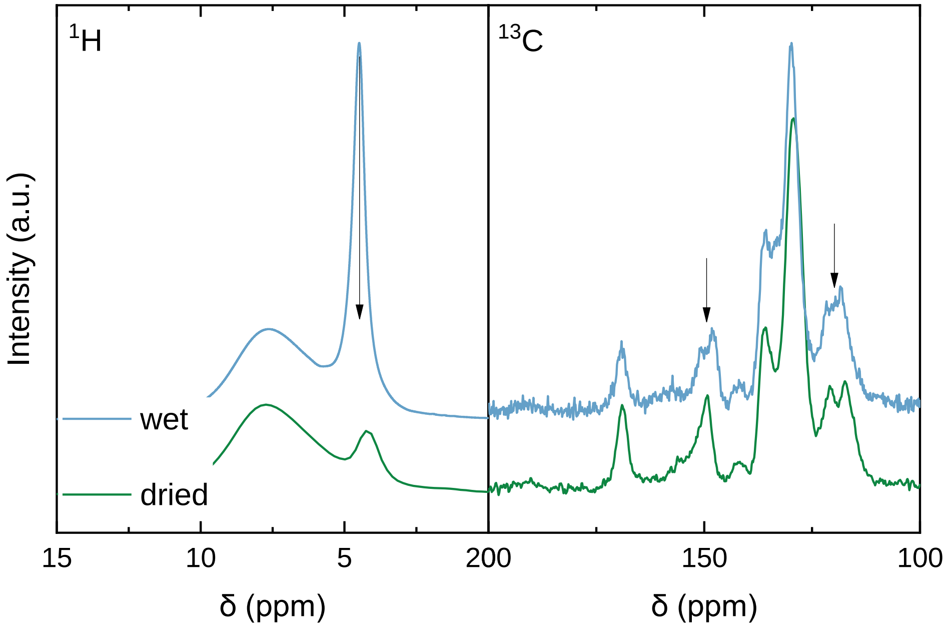

Figure S 18: ¹H and ¹³C-ssNMR comparison of wet, and dried NO-PI-3-COF with only minor residual water adsorbed. Both spectra were recorded on the same sample before and after drying under high vacuum. Arrows serve as a guide to the eye and indicate regions with differences between both spectra.

Nitrogen Gas Sorption Experiments

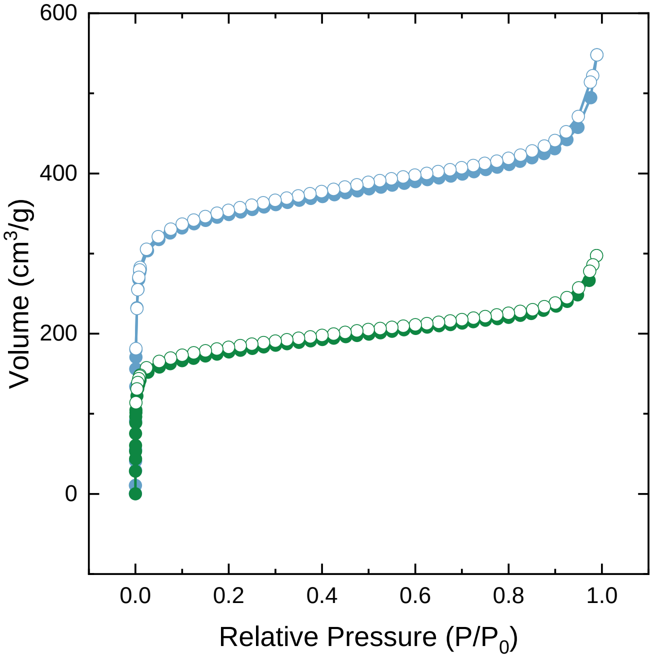

Figure S 19: N_2 sorption isotherms of PI-3-COF (blue) and NO-PI-3-COF (green), synthesized by direct oxidation of PI-3-COF. Filled dots represent data points of the adsorption branch, hollow dots those of the desorption branch, respectively.

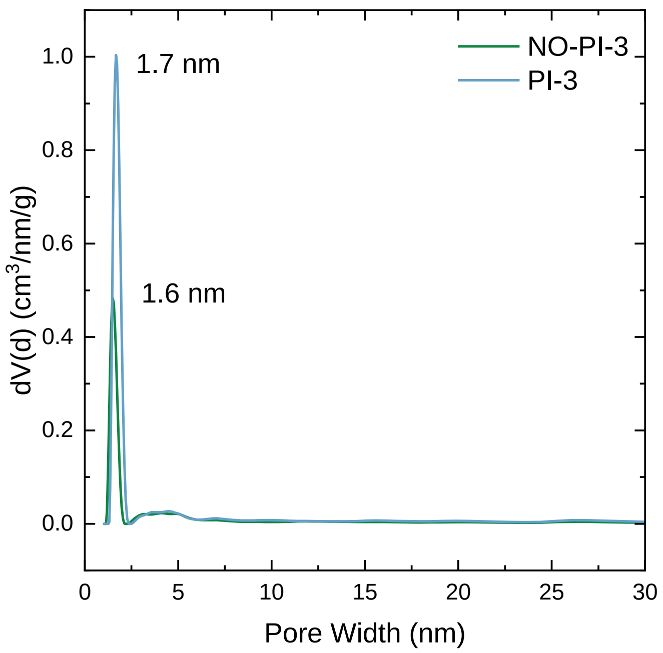

Figure S 20: Pore-size distribution of PI-3-COF and NO-PI-3-COF.

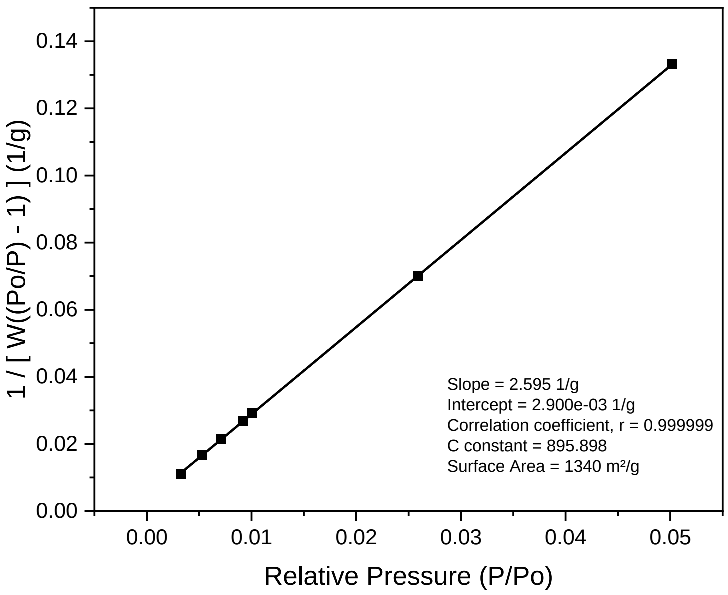

Figure S 21: BET plot for PI-3-COF.

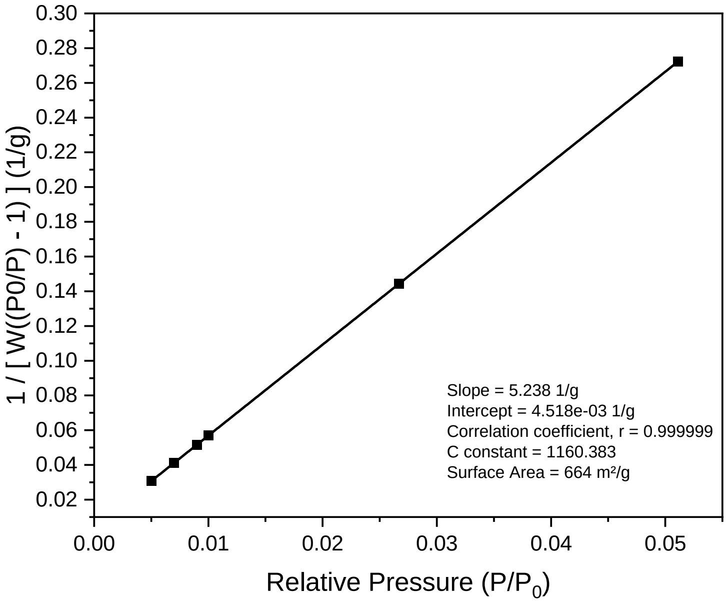

Figure S 22: BET plot for NO-PI-3-COF.

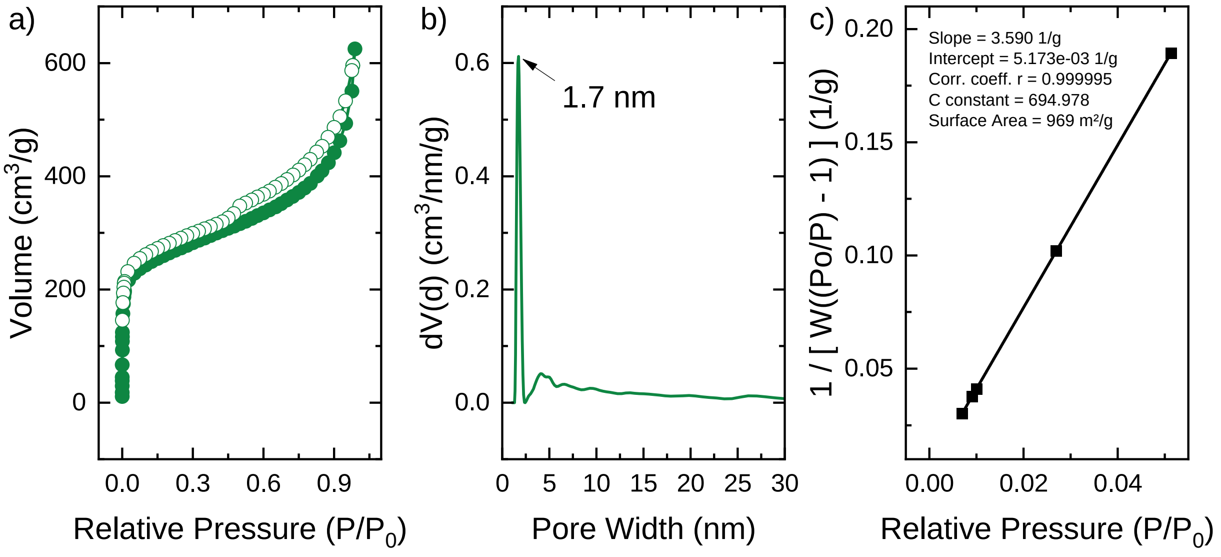

Figure S 23: N_2 sorption isotherm (a), pore-size distribution (b), and BET plot (c) for rPI-3-COF.

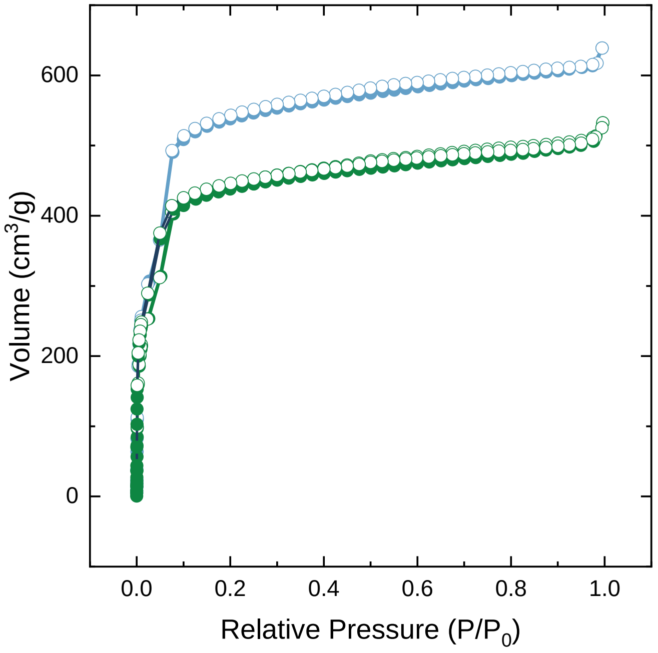

Figure S 24: N_2 sorption isotherms of TTI-COF (blue), rTTI-COF (green), and NO-TTI-COF (dark blue) are overlaid. Filled dots represent data points of the adsorption branch, hollow dots those of the desorption branch, respectively.

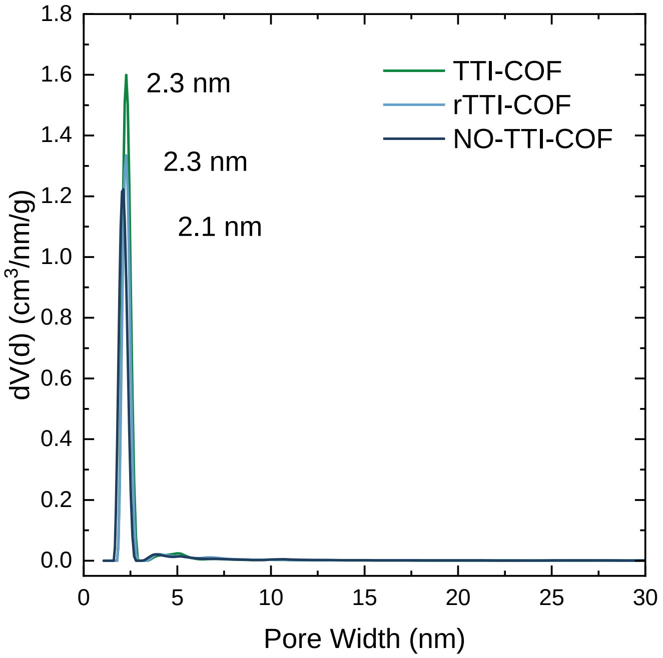

Figure S 25: Pore-size distribution of TTI-COF, rTTI-COF and NO-TTI-COF.

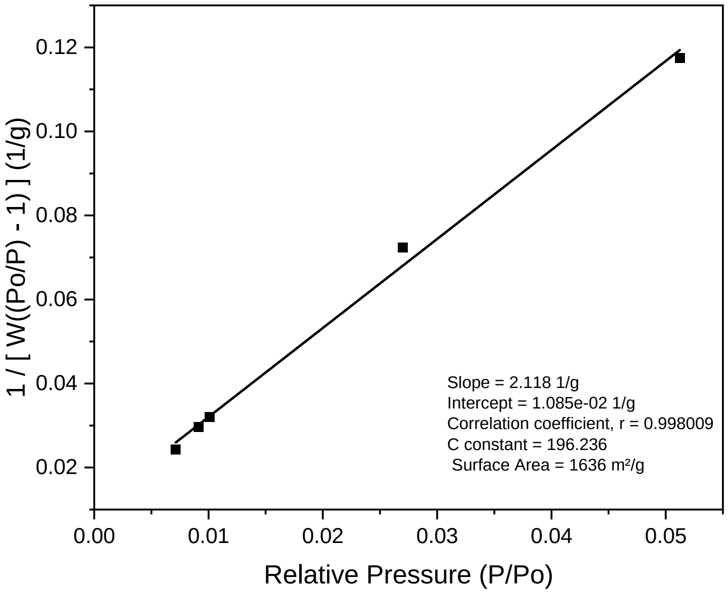

Figure S 26: BET plot for TTI-COF.

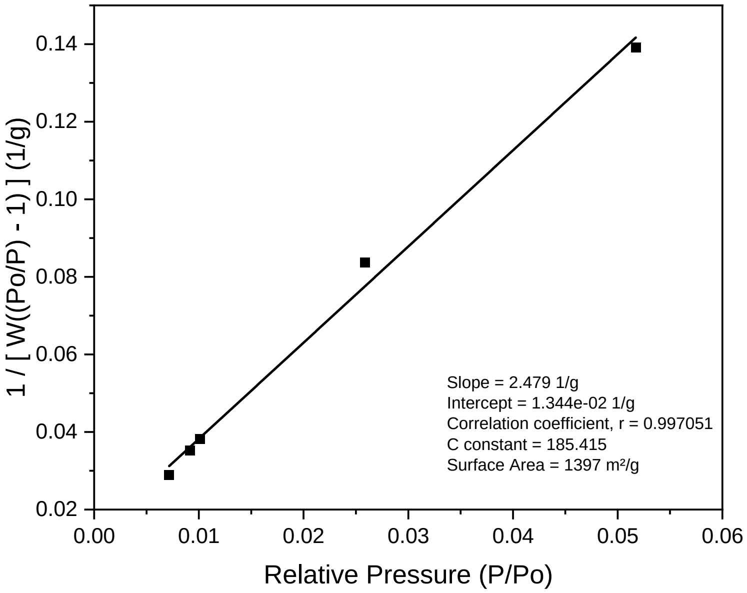

Figure S 27: BET plot for rTTI-COF.

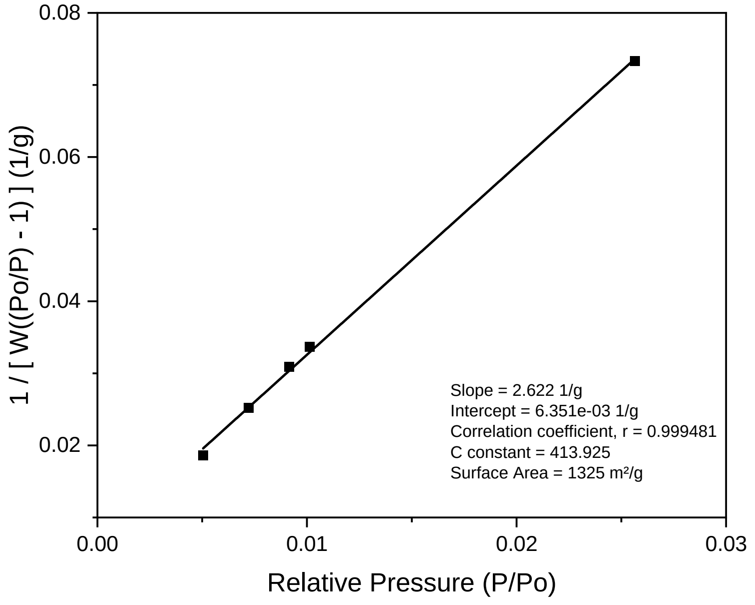

Figure S 28: BET plot for NO-TTI-COF.

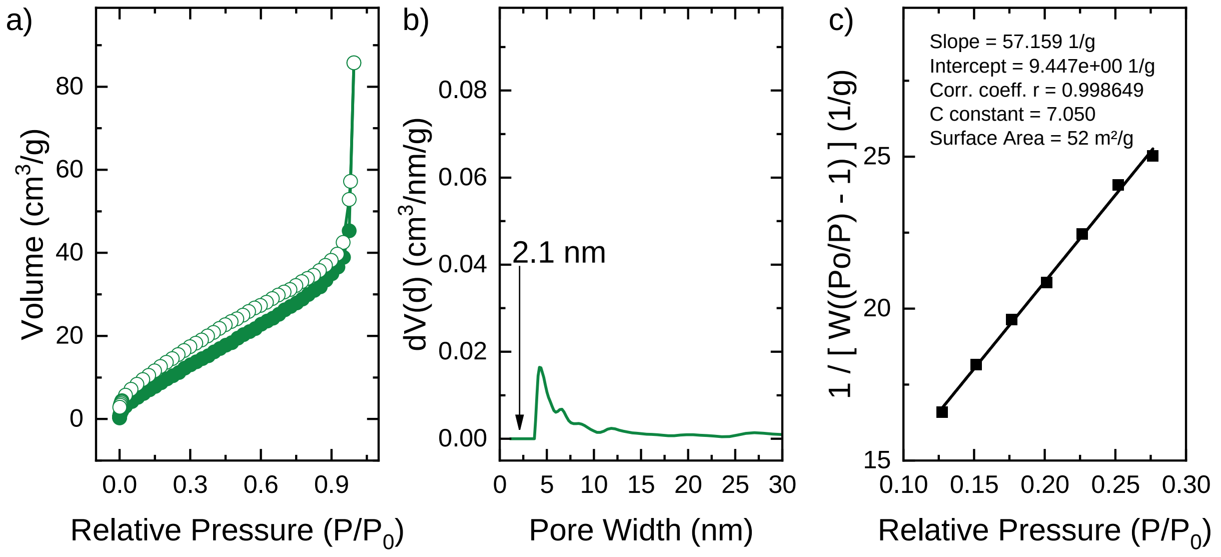

Figure S 29: N_2 sorption isotherm (a), pore-size distribution (b), and BET plot (c) for NO-TTI-COF after water adsorption. The data shows that the initial porosity of the material is lost due to drying-induced collapse of the 2.1 nm pore channels. Only a small fraction of textural porosity remains, which is responsible for a comparably small residual surface area.

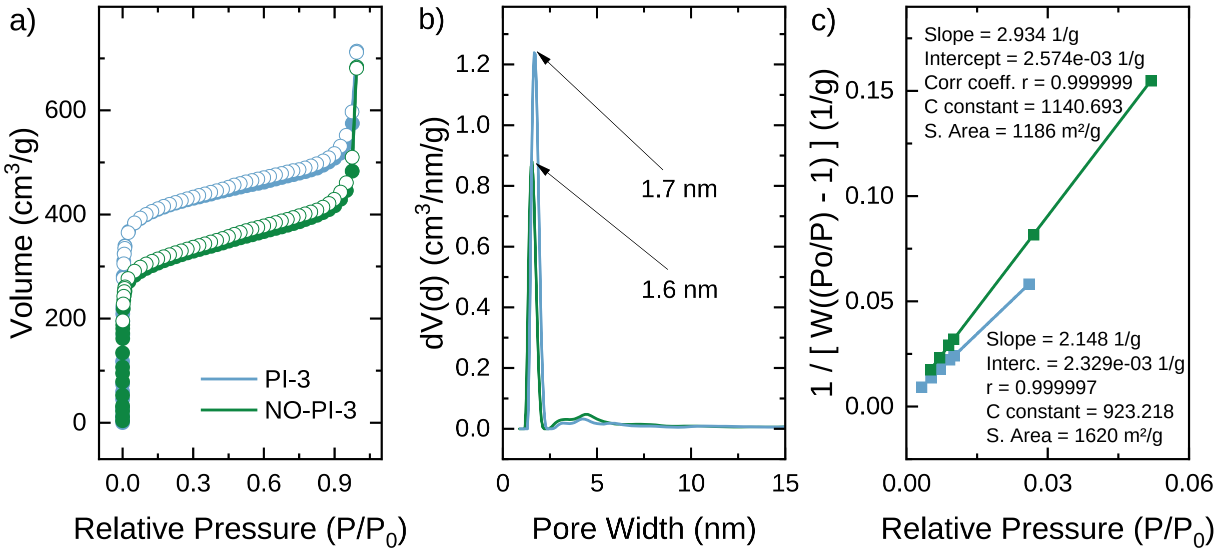

Figure S 30: N_2 sorption isotherm (a), pore-size distribution (b), and BET plot (c) for PI-3-COF and NO-PI-3-COF obtained via direct oxidation of PI-3-COF (reproduction experiment). NO-PI-3-COF was treated with $scCO_2$ and residual adsorbed water was removed by storing the sample in a desiccator over $CaCl_2$. Compared to a previous sample (Figure S 19, heated under dynamic vacuum) the loss in porosity after oxidation of PI-3-COF was reduced and allowed to obtain a sample of NO-PI-3 COF with larger surface area of S_{BET} = 1186 m^2g^{-1}.

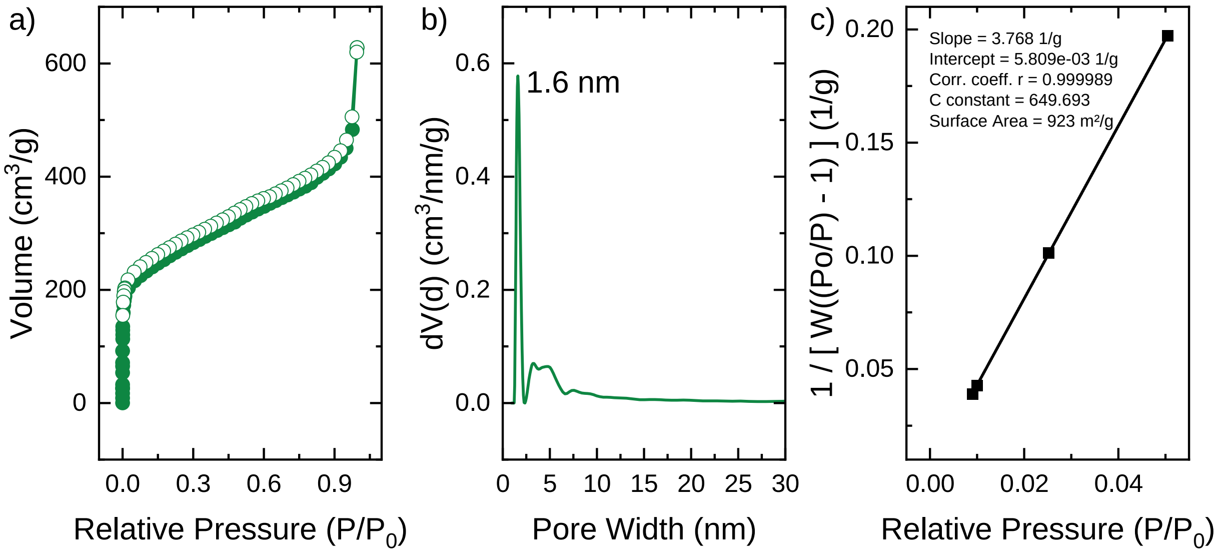

Figure S 31: N_2 sorption isotherm (a), pore-size distribution (b), and BET plot (c) for NO-PI-3-COF after in situ XRPD experiments with humidity control. Compared to the initial value (S_{BET} = 1186 m^2g^{-1}) the surface area of the material is slightly reduced, which is in contrast to the observed cycling performance (Figure S 56) and long-term moisture stability (Figure S 9) of the material. Note that the amount of sample available for the analysis was only ~3 mg, due to the limited capacity of the sample holder (see Figure S 53c), which might impact the experimental and weighting accuracy. In addition, remaining moisture after the water vapor desorption might have caused stress on the material during the heated vacuum treatment performed prior to the N_2 adsorption measurement.

Water Vapor Sorption Experiments

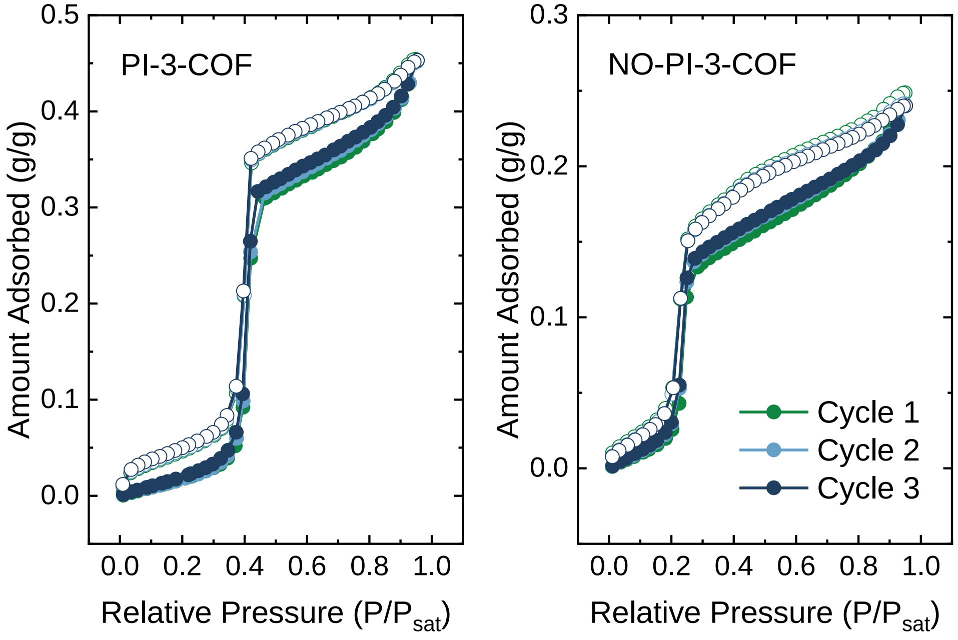

Figure S 32: Water vapor sorption isotherms of three consecutive cycles measured at T = 25°C for PI-3-COF and NO-PI-3-COF. Within the experimental error, no reduction in uptake capacity within these cycles is observed.

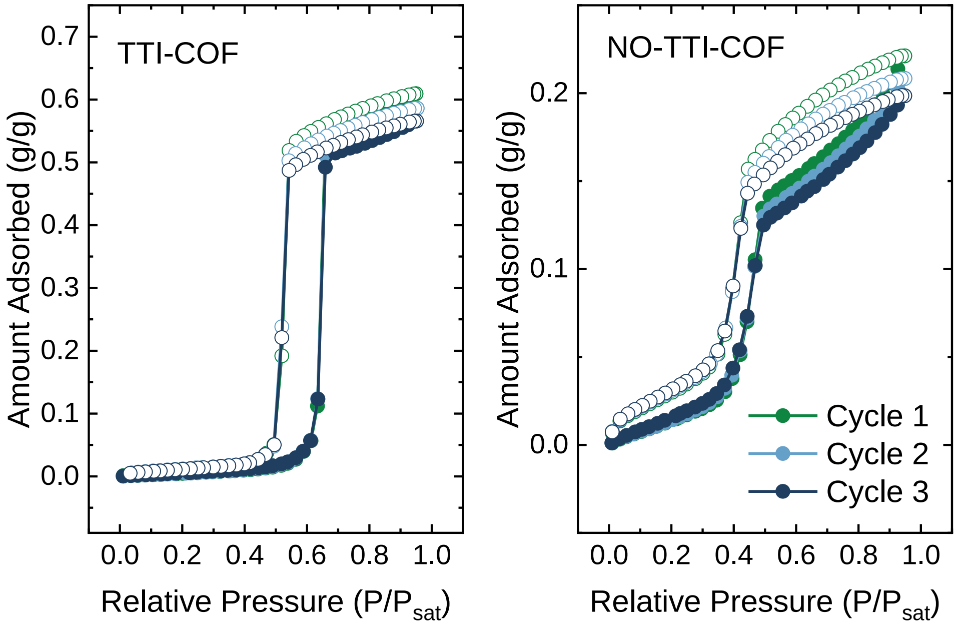

Figure S 33: Water vapor sorption isotherms of three consecutive cycles measured at T = 25°C for TTI-COF and NO-TTI-COF. A small but minor gradual decrease in maximum uptake capacity within these cycles is observed. Cycling measurements were performed after variable temperature measurements presented in Figure S 35.

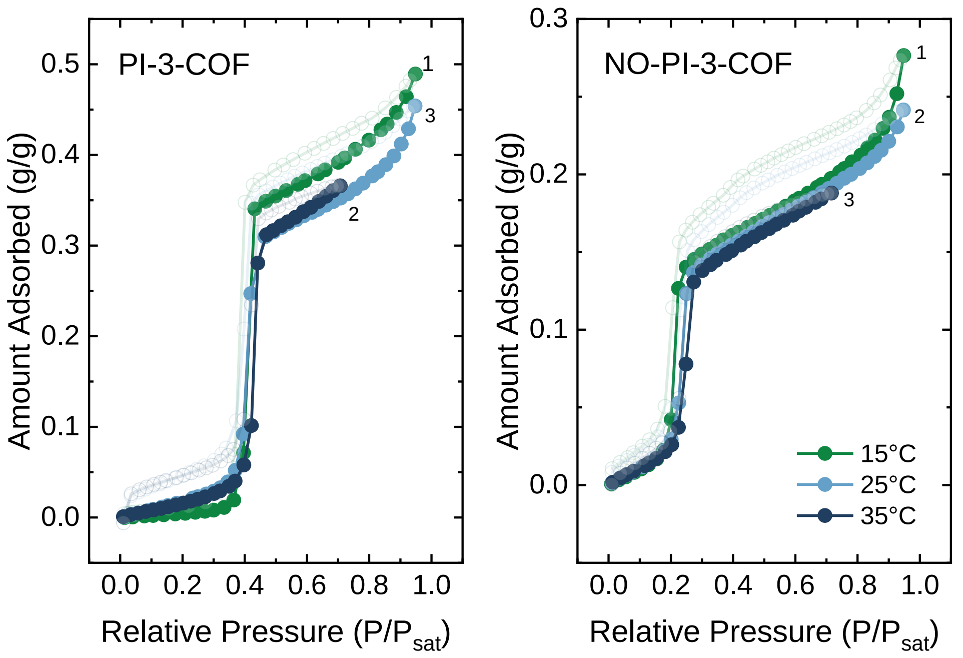

Figure S 34: Water vapor sorption isotherms of PI-3-COF and NO-PI-3-COF at different temperatures. Numbering denotes the order of measurement.

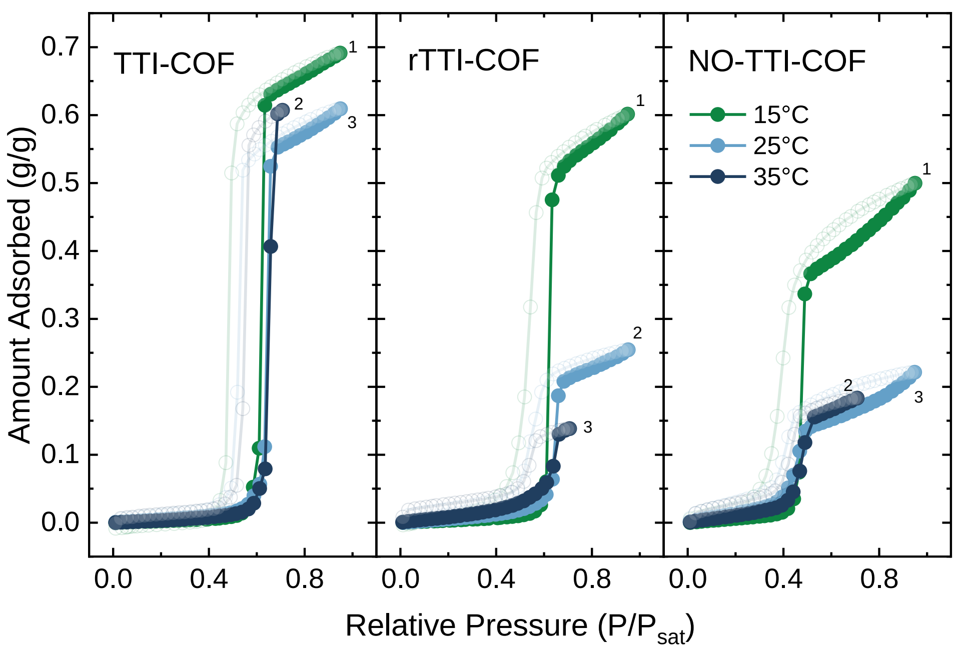

Figure S 35: Water vapor sorption isotherms of TTI-COF, rTTI and NO-TTI-COF at different temperatures. Numbers next to the isotherm denote the order of measurement. A major reduction in uptake capacity is visible for the second measurement of rTTI- and NO-TTI-COF, following the order of measurement. This indicates that the pore volume decreases after each adsorption-desorption cycle, which is related to a collapse of the pores in the material.

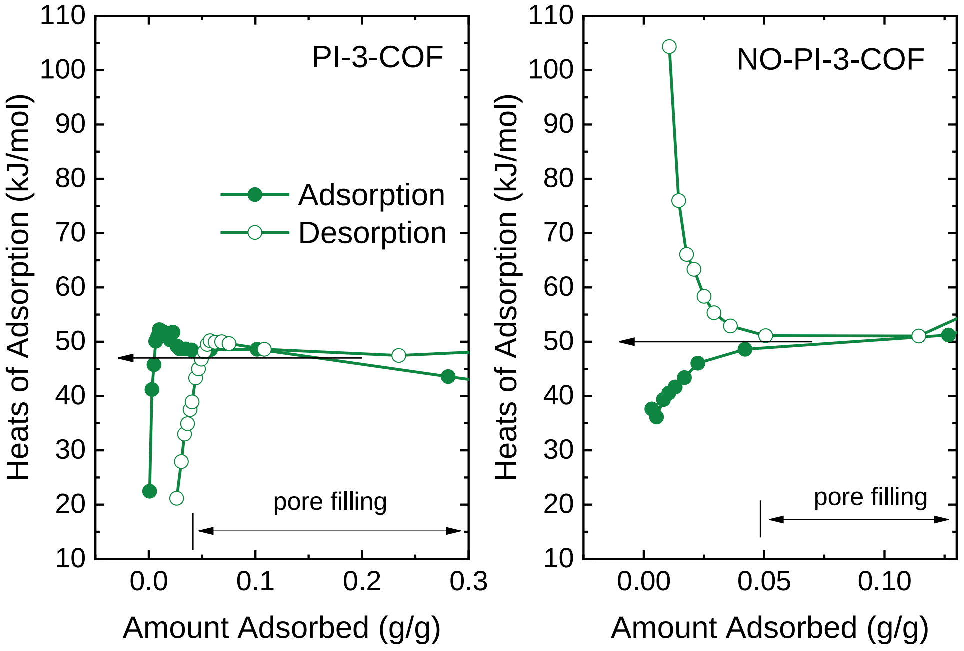

Figure S 36: Isosteric heats of adsorption at small loadings of adsorbed water vapor, approaching the pore condensation step, in PI-3-COF and NO-PI-3-COF. Values were calculated from vapor sorption isotherms at different temperatures. Note that calculated values at small loadings are associated with a larger error, caused by limited measurement accuracy, as well as kinetic influences during the sorption process (e.g. hysteresis) differing from ideal conditions.

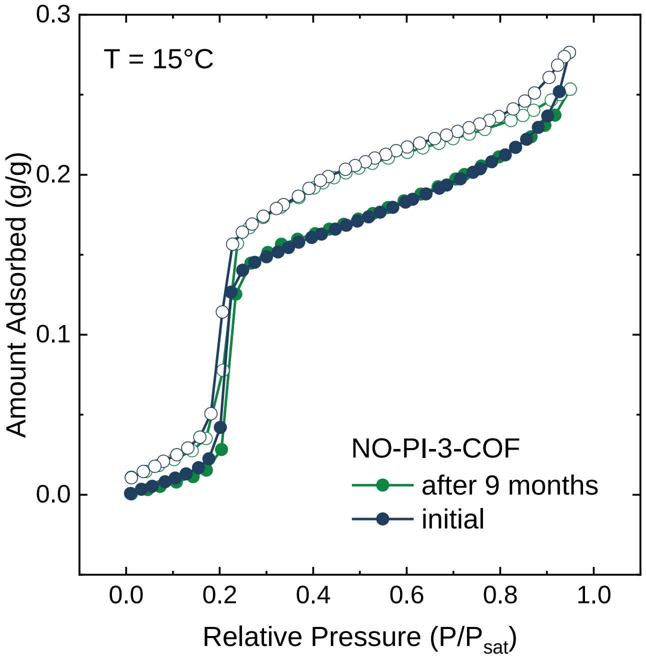

Figure S 37: Water vapor adsorption isotherms of NO-PI-3-COF at 15°C before and after storing the sample under ambient conditions i.e. in the hydrated state. The isotherms show a negligible difference indicating long-term stability of the material under exposure to moisture, which is in line with the cycling stability experiments presented in Figure S 32.

Table S 2: Comparison of reported COF water uptake capacities at low relative humidity.

COF	Water uptake (g/g) at P/P_{sat} = 0.3 @ 298K	BET surface area (m^2/g)	Linkage	Synthesis	Reference
AB-COF	0.34	1209	azine	de-novo	19
COF-480 hydrazide	0.33	989	azine/hydrazide	PSM of azine	19
TpPa-1	0.30	984	keto-enamine	de-novo	20
DHTa-Pa	0.49	2099	imine/keto-enamine	de-novo	21
NO-PI-3	0.15<br>0.27 (288K)	664<br>1186	nitrone	PSM of imine	This work

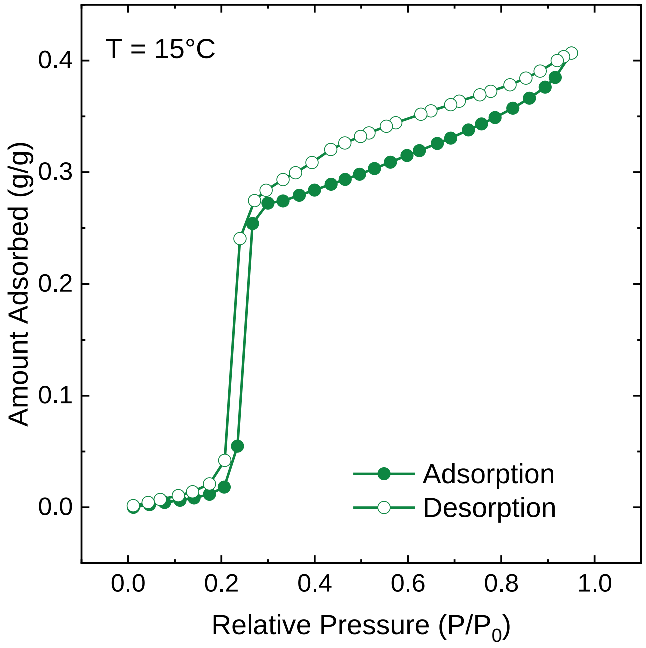

Figure S 38: Water vapor adsorption isotherms of NO-PI-3-COF (reproduction) at 15°C. This sample was obtained with larger BET surface area by careful drying in a desiccator to avoid vacuum induced stress (see Figure S 30). The increased porosity also results in a higher water uptake capacity.

CO_2 Gas Sorption Experiments

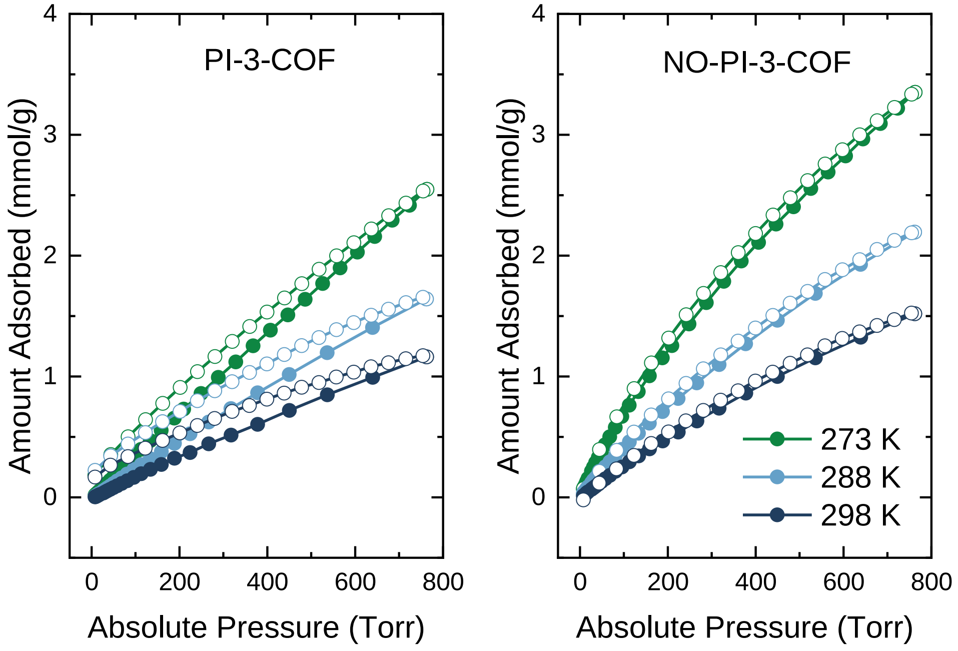

Figure S 39: CO_2 sorption isotherms of PI-3 and NO-PI-3-COF at different temperatures. Filled dots represent data points of the adsorption branch, hollow dots those of the desorption branch, respectively.

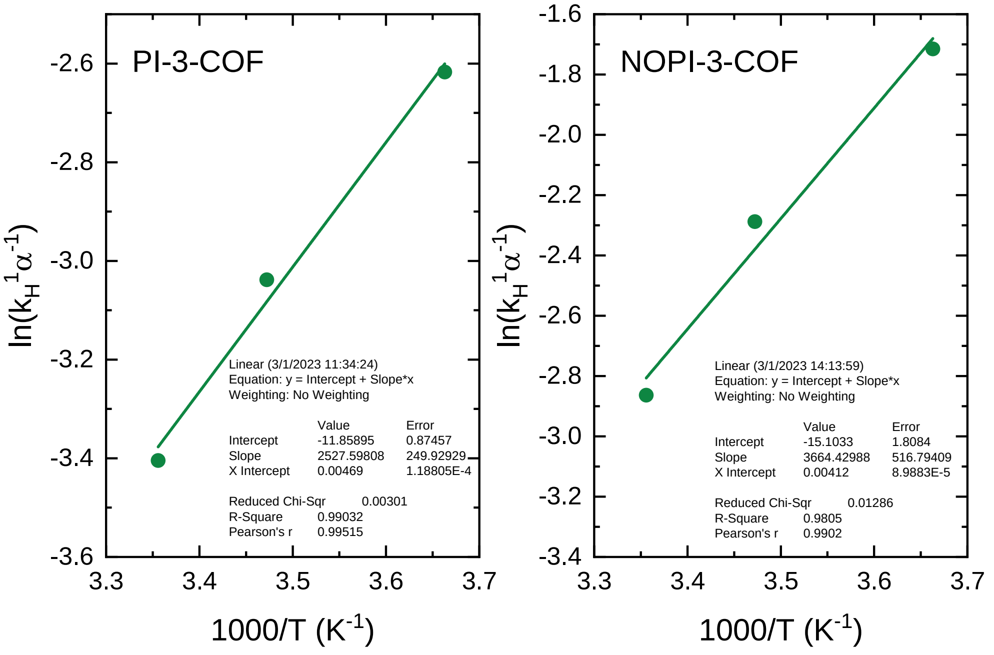

Figure S 40: Arrhenius plot of $k_H^1\alpha^{-1}$, obtained by a linear fit of the CO_2 adsorption isotherms at low pressures for PI-3- and NO-PI-3-COF vs. temperature. Slopes of the linear fits allow to estimate ΔQ_{st} at zero coverage (see methods for details) and give values of ΔQ_{st} = 21 ± 2 kJ^1mol^{-1} (PI-3-COF) and ΔQ_{st} = 30 ± 4 kJ^1mol^{-1} (NO-PI-3-COF).

Thermogravimetric Analysis

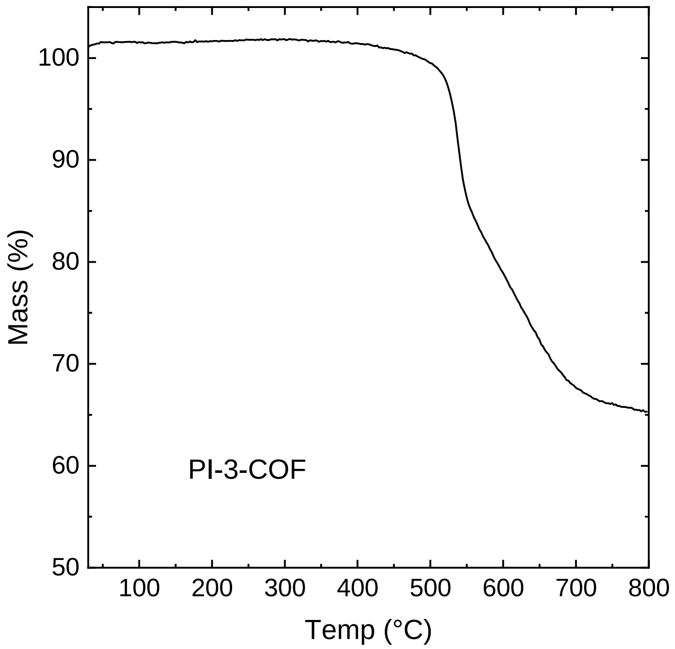

Figure S 41: Thermogravimetric trace of PI-3-COF under argon flow.

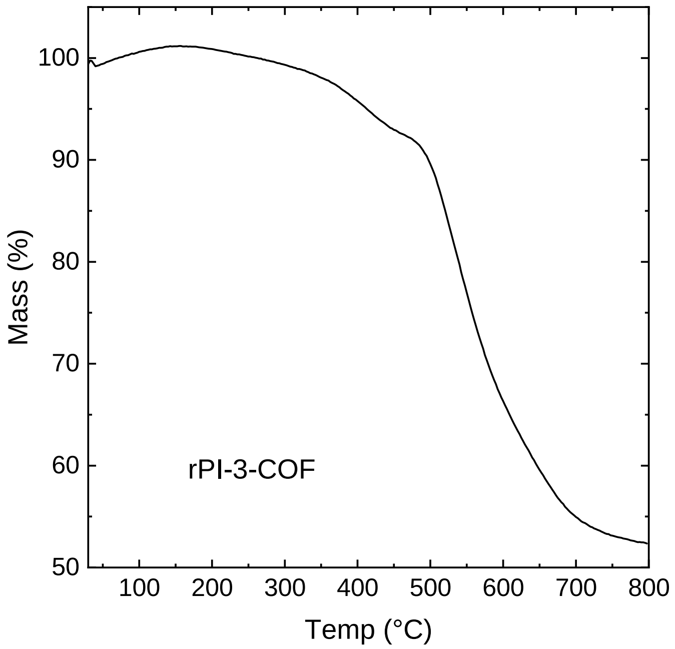

Figure S 42: Thermogravimetric trace of rPI-3-COF under argon flow.

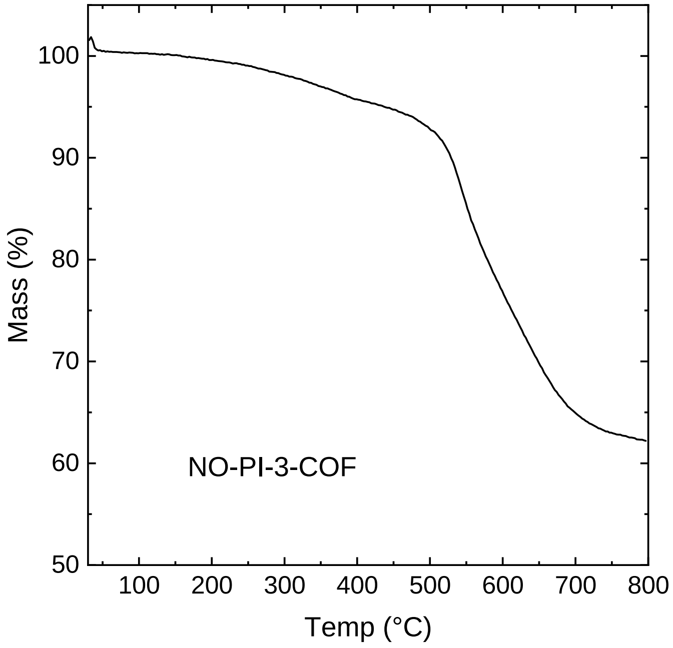

Figure S 43: Thermogravimetric trace of NO-PI-3-COF under argon flow.

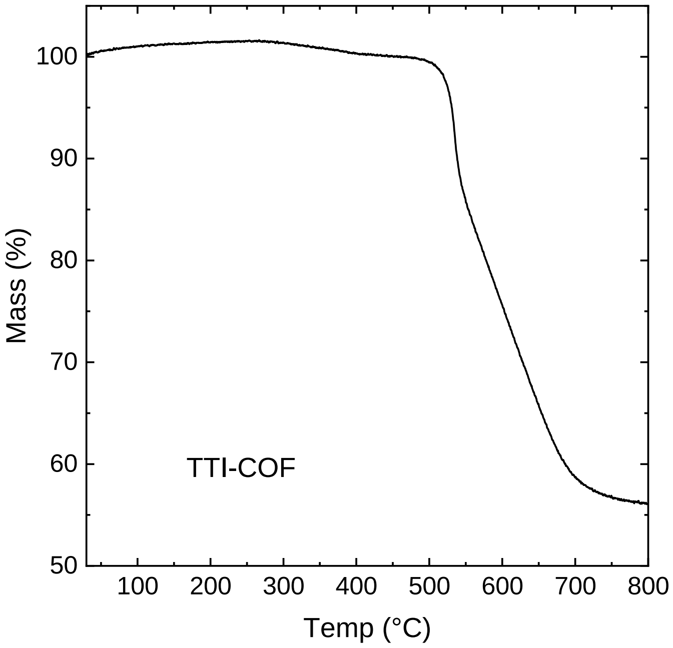

Figure S 44: Thermogravimetric trace of TTI-COF under argon flow.

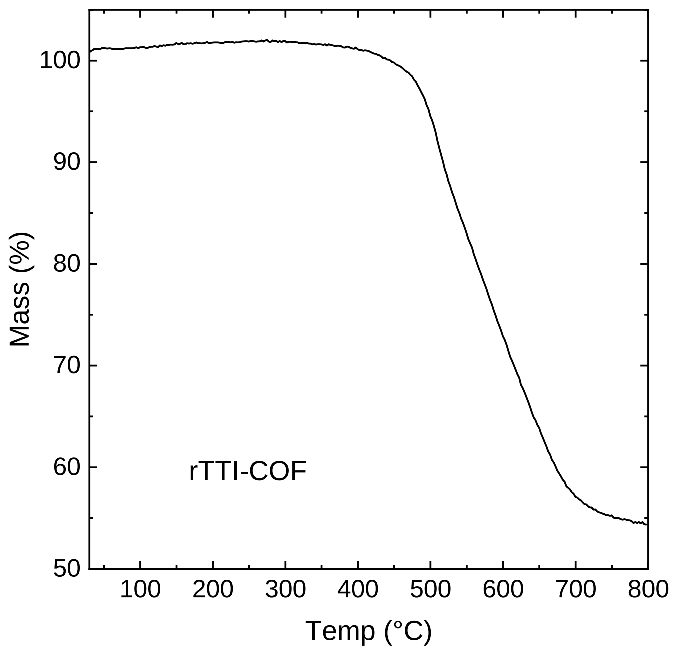

Figure S 45: Thermogravimetric trace of rTTI-COF under argon flow.

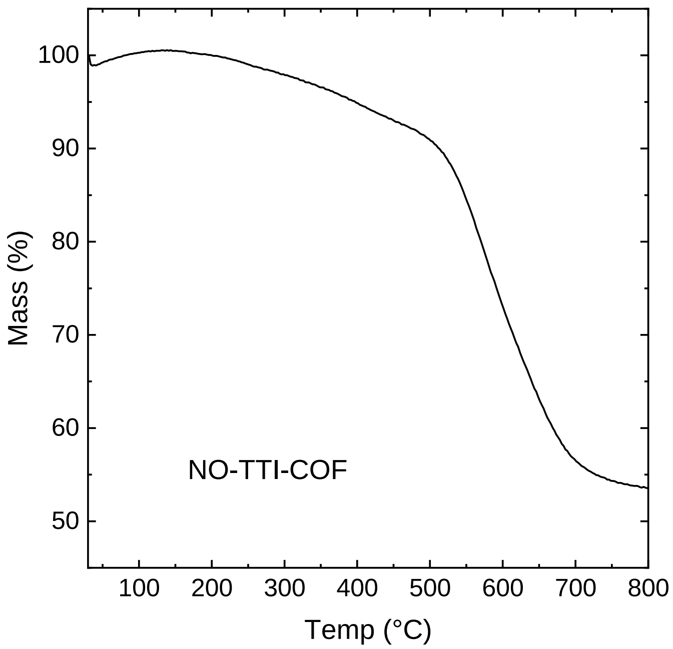

Figure S 46: Thermogravimetric trace of NO-TTI-COF under argon flow.

Quantum Chemical Calculations

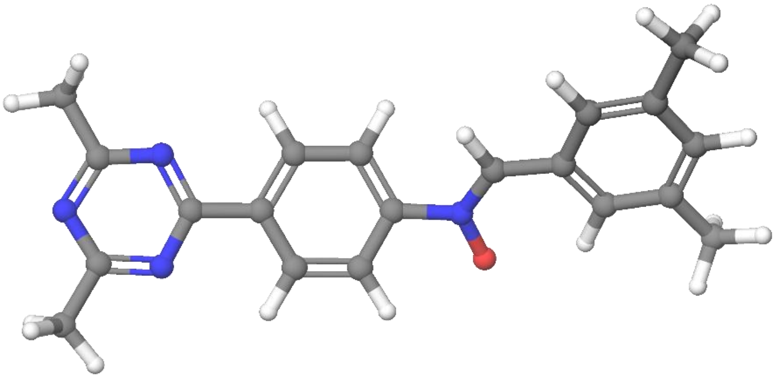

Figure S 47: Optimized structure for a representative molecular model system of NO-PI-3-COF (NO-PI-3 M), obtained on PBE0-D3/def2-TZVP level of theory.

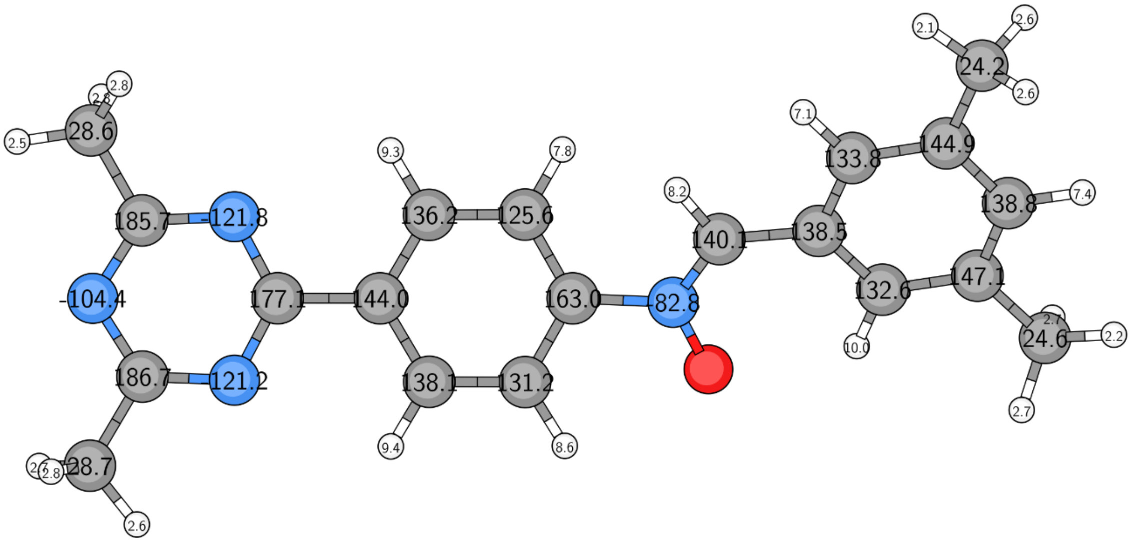

Figure S 48: Calculated NMR Chemical Shifts for the NO-PI-3 M model system, obtained on B97-2/pcS-2//PBE0-D3/def2-TZVP level of theory.

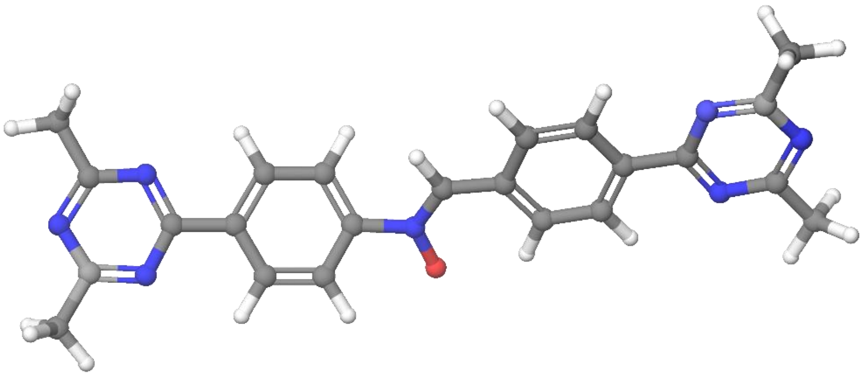

Figure S 49: Optimized structure for a representative molecular model system of NO-TTI-COF (NO-TTI M), obtained on PBE0-D3/def2-TZVP level of theory.

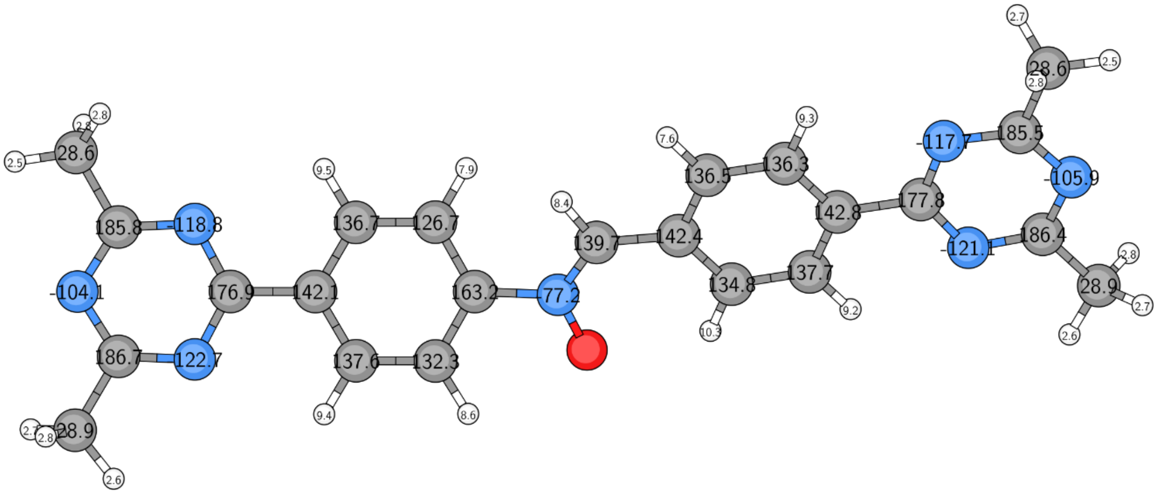

Figure S 50: Calculated NMR Chemical Shifts for the NO-TTI M model system, obtained on B97-2/pcS-2//PBE0-D3/def2-TZVP level of theory.

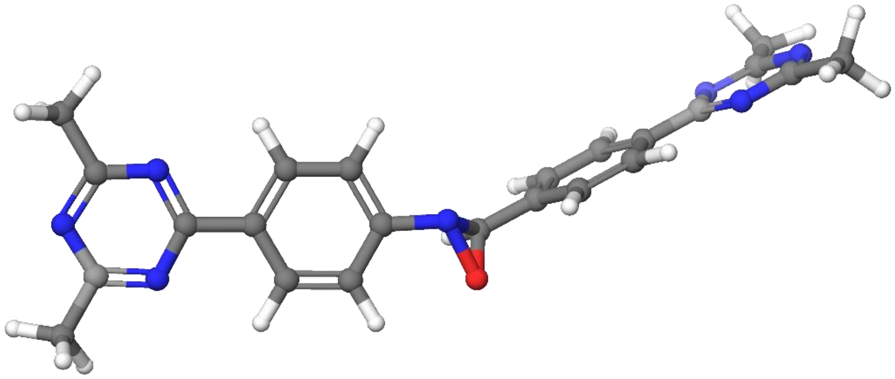

Figure S 51: Optimized structure for a theoretical oxaziridine-linkage in the TTI M model system, obtained on PBE0-D3/def2-TZVP level of theory.

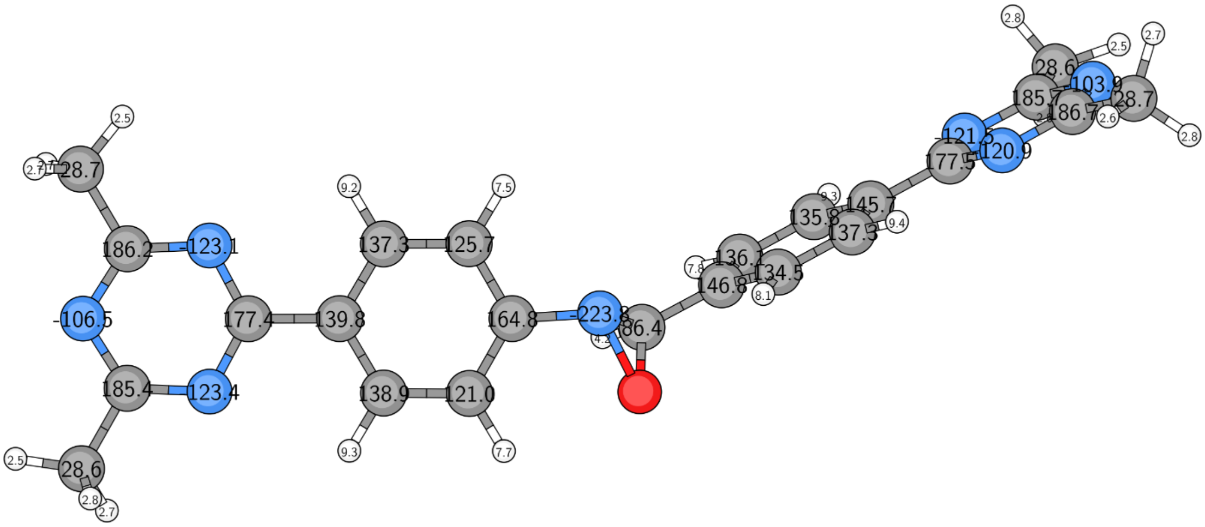

Figure S 52: Calculated NMR Chemical Shifts for a theoretical oxaziridin-linkage in the TTI M model system, obtained on B97-2/pcS-2//PBE0-D3/def2-TZVP level of theory. The absence of aliphatic carbon signals at ~86 ppm in the experimental carbon ssNMR spectrum excludes the formation of this linkage in the COF.

In situ X-ray powder diffraction:

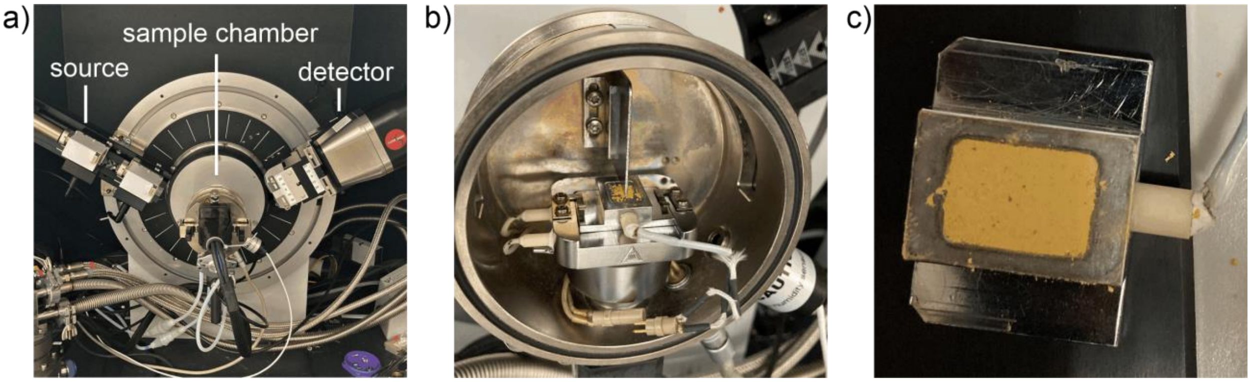

Figure S 53: Instrumental setup (a) used for in situ XRPD experiments at controlled humidity. The humidity and temperature of the sample chamber (b) is controlled during the measurement. The sample (NO-PI-3-COF) is spread-out on the flat sample holder (c) to allow vapor exchange with the humidified nitrogen gas stream.

XRPD patterns under humidity controlled atmosphere were collected using a Bruker D8 advanced diffractometer using Cu-$K_{\alpha 1}$ radiation from a Johann-type Ge111 monochromator and a Lynx Eye detector (Bruker) equipped with a humidity chamber (Anton Paar). The humidity within the chamber was adjusted by mixing a dry and a water vapor saturated nitrogen stream. A total flow rate of 500 mL/min was applied and the temperature of the chamber was constantly kept at 25.0 ± 0.2 °C. NO-PI-3-COF was exposed to 0.1 and 40 % r.H for switching between the dehydrated and hydrated state. For measurements on the hydration and dehydration kinetics a total scan time of 10 minutes was used. The cycling behavior was monitored by exposing NO-PI-3-COF to the "dry" (0.1 % R.H.) and "wet" state (40 % R.H.) for one hour per step. A delay time of 40 minutes, prior to a measurement using a scan time of 20 minutes was applied. The XRPD data analyses was performed using TOPAS v6.[22]

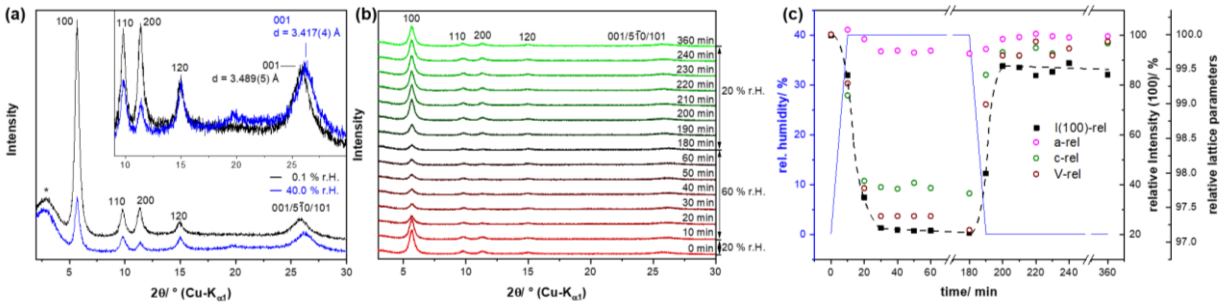

Figure S 54: In situ XRPD measurements NO-PI-3-COF (a): XRPD patterns in dehydrated (black lines) and hydrated states R.H. (blue lines) including the diffraction signal attributed to the empty sample holder and the humidity chamber (grey line, asterisks) and selected reflection indices, (b) time dependent in situ XRPD patterns during hydration and dehydration, (c) quantitative analyses of the diffraction patterns during hydration and dehydration.

Upon hydration, the diffraction signals of the COF seem to decrease in intensity (Figure S 54a, blue lines). A close inspection of XRPD patterns, however, reveals that only certain reflections like 100, 110 or 200 become weaker, whereas the intensity of other reflections like 120 stays constant (inset). As the peaks do not become broader, these diffraction effects are attributed to the incorporation of water into the crystal structure (leading to a reduced scattering contrast) rather than to a loss of the crystallinity of the COF (see

below). Moreover, the incorporation of water into the structure leads to a significant upshift of the position of the 001 reflection, which corresponds to a decrease of the *c*-lattice parameter from 3.489(5) Å to 3.417(4) Å. When the relative humidity is subsequently decreased, the peak intensities of 100, 110 or 200 increase again (Figure S 54b, c), which shows that the hydration of the COFs is reversible. We conducted repeated measurements to gain insights into the de- and rehydration kinetics: the change in peak intensities upon de- and rehydration was observed to occur within 30-40 minutes (Figure S 54b). At the end of the re- and dehydration cycle, the intensities do not fully revert to their initial state, which can be depicted best by monitoring the evolution of the 100 reflection intensity (Figure S 54c, black squares). After dehydration, the intensity of the 100 reflection only reaches 85 % of its original value, which suggests that after dehydration some water remains in the pores, even at lower relative humidity. The quality of the diffraction data of the NO-PI-3-COF allowed us to perform a more in depth analyses by fully weighted Rietveld[23] refinements (Figure S 54c) for tracking the evolution of the lattice parameters. Hydration of the NO-PI-3-COF leads to a significant reduction of the unit cell volume by more than 2.5 % (brown circles), mainly driven by the contraction of the *c*-lattice parameter of more than 2 % (green circles), whereas the *a*-axis only slightly shortens by < 0.5 % (magenta circles). In conclusion, an uptake of water leads to a contraction of the mean interlayer distance and therefore to a contraction of the unit cell. This might appear counterintuitive, however by filling the pores with water molecules, the interlayer interactions can be increased mediated by hydrogen bonds among water molecules and neighboring COF layers. In addition, the water incorporation could trigger a conformational change of linker related groups and therefore lead to a more efficient packing of the COF layers.

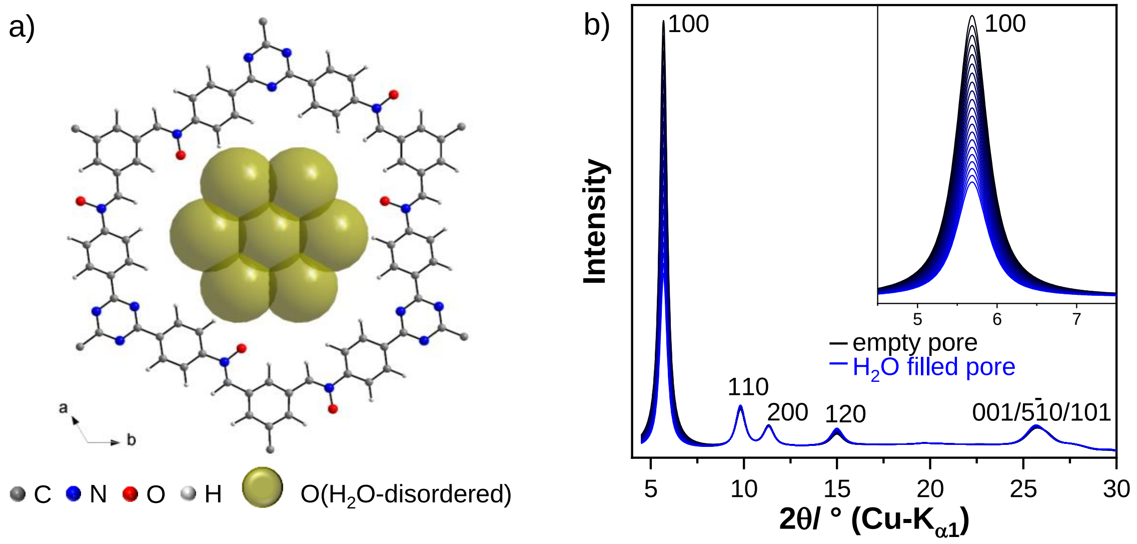

Figure S 55: Structure model of (a) NO-PI-3-COF filled with seven disordered water molecules per layer represented by oxygen atoms with artificially large thermal displacement parameters (large green balls), series of simulated XRPD patterns of NO-PI-3-COF, and (b) with an incremental increase of pore content starting with empty pores and extending to complete filling.

All XRPD data analyses were performed by fully weighted Rietveld[23] refinements using idealized models for the COF structures with planar layer and all possible torsion angles of the linker components fixed. The filling of the pores by disordered water molecules was simulated using oxygen atoms with artificially high thermal displacement parameters in a closed packed arrangement (Figure S 55a, green balls). For the NO-

PI-3-COF one pore was filled with seven water molecules per pore and layer. In order to visualize the impact of the pore filling on the diffraction patterns, we performed systematic simulations, where we calculated the XRPD patterns while incrementally increasing the occupancy of the pore water related oxygen sites from 0 (Figure S 55b, black lines) to 1 (blue lines), simulating a change from empty to water filled pores. An increasing pore content has significant impact on the XRPD pattern, especially on the 100 reflection, which shows a significant decrease in intensity. This decrease in peak intensity corresponds to the interaction of the increasing amount of diffuse electron density in the pores with the electron density of the COF-framework. All other reflection are not or only effected to lesser extent by this effect. It must be noted that an anisotropic occupation of the possible sites for pore water can change the magnitude of the decrease in the 100 peak intensity and can change the effect on other reflections. For the NO-PI-3-COF a significant decrease of the 110 and 200 peak intensities was noticed upon pore filling, whereas the intensity of the 120 stayed constant. Simulations with an isotropic pore filling, the intensity of the 110 and 200 diffraction lines hardly changes. This can point to an anisotropic filling of the pore in the real structure. In the simulations, the magnitude of the decrease in peak intensity was also impacted by the size of the isothermal displacement parameter that we chose arbitrarily. Given this and the possible change of the stacking order, degree of stacking faulting and torsion of the linkers of the COF framework, we decided to not use this model for quantitative analyses of the pore filling, i.e. for tracking the occupancy of the pore water related oxygen sites.

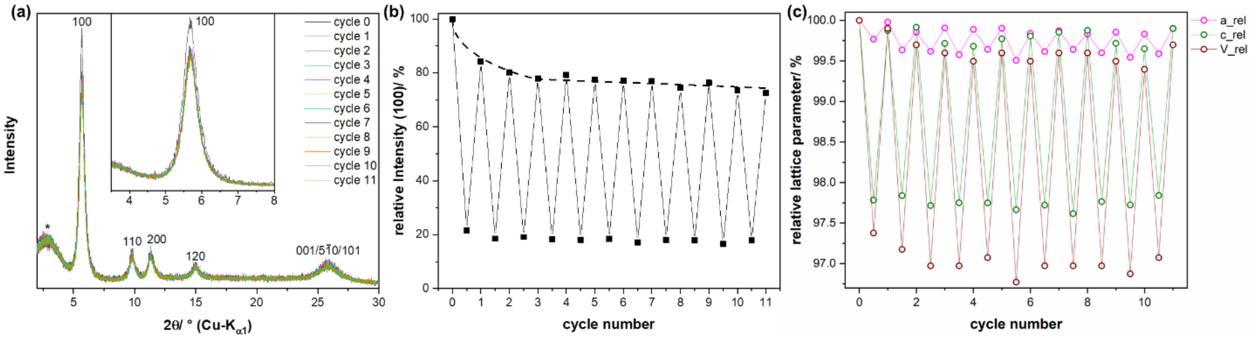

Figure S 56: XRPD patterns of NO-PI-3-COF (a) collected after different hydration-dehydration cycles, (b) evolution of the 100 peak intensities upon reversible hydration and dehydration, the dashed line visualizes the trend of the 100 peak intensity at the end of each cycle in dehydrated state, (c) evolution of the 100 peak intensity upon pore filling in the simulated XRPD patterns, (c) evolution of the unit cell volume and the a- and c-lattice parameter of the NO-PI-3-COF during hydration-dehydration cycles.

The reversible hydration and dehydration behavior of the COF material was monitored by in situ XRPD. Each hydration and dehydration cycle was started at 0.1 % R.H. Afterwards the humidity was ramped up to 40 % R.H. within one minute, kept constant, and subsequently the humidity was ramped down within one minute again. For every step the relative humidity was kept constant for one hour, which corresponds to a total cycle-length of two hours. The XRPD patterns reveal that the relative peak intensity (100), corresponding to the “dry” state of the COF, decreases during the first 3 cycles. This decrease is most pronounced after completion of the first hydration-cycle (Figure S 56b) and then the peak intensity stays constant upon continued hydration-dehydration cycles. The 100 reflection intensity in the dehydrated state stabilizes after four cycles at ≈ 75 % of its initial value (Figure S 56b, black dashed line), whereas the corresponding intensity in hydrated state stays constant at around 20 % throughout the entire experiment. This trend is also reflected by the evolution of the lattice parameters and of the unit cell

volume (Figure S 56c). These data show, that after the completion of the hydration-dehydration cycles, water remains in the material that is not easily desorbed by a simple reduction of the relative humidity. On the other hand the constant intensity of the 100 reflection for the hydrated state signifies that the material reaches the same level of hydration in each adsorption cycle – and thus remains chemically and structurally intact throughout the cycles. This conclusion is also in agreement with the observed cycling stability during the presented volumetric water vapor adsorption experiments.

References

1. Perdew J. P., Burke K., Ernzerhof M. Generalized Gradient Approximation Made Simple. *Phys. Rev. Lett.* **77**, 3865-3868 (1996).

2. Grimme S., Antony J., Ehrlich S., Krieg H. A consistent and accurate ab initio parametrization of density functional dispersion correction (DFT-D) for the 94 elements H-Pu. *J. Chem. Phys.* **132**, 154104 (2010).

3. Schäfer A., Huber C., Ahlrichs R. Fully optimized contracted Gaussian basis sets of triple zeta valence quality for atoms Li to Kr. *J. Chem. Phys.* **100**, 5829-5835 (1994).

4. Eichkorn K., Weigend F., Treutler O., Ahlrichs R. Auxiliary basis sets for main row atoms and transition metals and their use to approximate Coulomb potentials. *Theor. Chem. Acc.* **97**, 119-124 (1997).

5. Burow A. M., Sierka M. Linear Scaling Hierarchical Integration Scheme for the Exchange-Correlation Term in Molecular and Periodic Systems. *J. Chem. Theory Comput.* **7**, 3097-3104 (2011).

6. Burow A. M., Sierka M., Mohamed F. Resolution of identity approximation for the Coulomb term in molecular and periodic systems. *J. Chem. Phys.* **131**, 214101 (2009).

7. Grajciar L. Low-memory iterative density fitting. *J. Comput. Chem.* **36**, 1521-1535 (2015).

8. Lazarski R., Burow A. M., Grajciar L., Sierka M. Density functional theory for molecular and periodic systems using density fitting and continuous fast multipole method: Analytical gradients. *J. Comput. Chem.* **37**, 2518-2526 (2016).

9. Łazarski R., Burow A. M., Sierka M. Density Functional Theory for Molecular and Periodic Systems Using Density Fitting and Continuous Fast Multipole Methods. *J. Chem. Theory Comput.* **11**, 3029-3041 (2015).

10. TURBOMOLE V7.3 2018, a development of University of Karlsruhe and Forschungszentrum Karlsruhe GmbH, 1989-2007, TURBOMOLE GmbH, since 2007; available from http://www.turbomole.com.

11. Adamo C., Barone V. Toward reliable density functional methods without adjustable parameters: The PBE0 model. *J. Chem. Phys.* **110**, 6158-6170 (1999).

12. Ernzerhof M., Scuseria G. E. Assessment of the Perdew–Burke–Ernzerhof exchange-correlation functional. *J. Chem. Phys.* **110**, 5029-5036 (1999).

13. Wilson P. J., Bradley T. J., Tozer D. J. Hybrid exchange-correlation functional determined from thermochemical data and ab initio potentials. *J. Chem. Phys.* **115**, 9233-9242 (2001).

14. Jensen F. Basis Set Convergence of Nuclear Magnetic Shielding Constants Calculated by Density Functional Methods. *J. Chem. Theory Comput.* **4**, 719-727 (2008).

15. Kussmann J., Ochsenfeld C. Preselective Screening for Linear-Scaling Exact Exchange-Gradient Calculations for Graphics Processing Units and General Strong-Scaling Massively Parallel Calculations. *J. Chem. Theory Comput.* **11**, 918-922 (2015).

16. Kussmann J., Ochsenfeld C. Pre-selective screening for matrix elements in linear-scaling exact exchange calculations. *J. Chem. Phys.* **138**, 134114 (2013).

17. Horn A., Kazmaier U. Purified mCPBA, a Useful Reagent for the Oxidation of Aldehydes. *Eur. J. Org. Chem.* **2018**, 2531-2536 (2018).

18. Grunenberg L. et al. Amine-Linked Covalent Organic Frameworks as a Platform for Postsynthetic Structure Interconversion and Pore-Wall Modification. *J. Am. Chem. Soc.* **143**, 3430-3438 (2021).

19. Nguyen H. L. et al. Hydrazine-Hydrazide-Linked Covalent Organic Frameworks for Water Harvesting. *ACS Cent. Sci.* **8**, 926-932 (2022).

20. Biswal B. P. et al. Pore surface engineering in porous, chemically stable covalent organic frameworks for water adsorption. *J. Mater. Chem. A* **3**, 23664-23669 (2015).

21. Sun C. et al. 2D Covalent Organic Framework for Water Harvesting with Fast Kinetics and Low Regeneration Temperature. *Angew. Chem. Int. Ed.* **62**, e202217103 (2023).

22. Coelho, A. A., TOPAS and TOPAS-Academic: an optimization program integrating computer algebra and crystallographic objects written in C++. *J. Appl. Crystallogr.* **2018,** 51 (1), 210-218.

23. Rietveld, H. M., A profile refinement method for nuclear and magnetic structures. *J. Appl. Crystallogr.* **1969,** 2 (2), 65-71.
